# Signature construction and molecular subtype identification based on liver-specific genes for prediction of prognosis, immune activity, and anti-cancer drug sensitivity in hepatocellular carcinoma

**DOI:** 10.1186/s12935-024-03242-3

**Published:** 2024-02-19

**Authors:** Xiuzhi Zhang, Zhefeng Xiao, Xia Zhang, Ningning Li, Tao Sun, JinZhong Zhang, Chunyan Kang, Shasha Fan, Liping Dai, Xiaoli liu

**Affiliations:** 1 Department of Pathology, Henan Medical College, Zhengzhou, 451191 Henan China; 2 Department of Pathology, NHC Key Laboratory of Cancer Proteomics, National Clinical Research Center for Geriatric Disorders, Xiangya Hospital, Central South University, Changsha, 410008 China; 3 Oncology Department, Key Laboratory of Study and Discovery of Small Targeted Molecules of Hunan Province, The First Affiliated Hospital of Hunan Normal University, Hunan Provincial People’s Hospital, Hunan Normal University, Changsha, 410000 Hunan China; 4 Henan Institute of Medical and Pharmaceutical Sciences, Zhengzhou University, Zhengzhou, 450052 China; 5 Laboratory Department, Henan Provincial People’s Hospital, Zhengzhou, 450003 China

**Keywords:** Liver-specific genes (LSGs), Hepatocellular carcinoma, Heterogeneity, Prognosis, Subtype

## Abstract

**Background:**

Liver specific genes (LSGs) are crucial for hepatocyte differentiation and maintaining normal liver function. A deep understanding of LSGs and their heterogeneity in hepatocellular carcinoma (HCC) is necessary to provide clues for HCC diagnosis, prognosis, and treatment.

**Methods:**

The bulk and single-cell RNA-seq data of HCC were downloaded from TCGA, ICGC, and GEO databases. Through unsupervised cluster analysis, LSGs-based HCC subtypes were identified in TCGA-HCC samples. The prognostic effects of the subtypes were investigated with survival analyses. With GSVA and Wilcoxon test, the LSGs score, stemness score, aging score, immune score and stromal score of the samples were estimated and compared. The HCC subtype-specific genes were identified. The subtypes and their differences were validated in ICGC-HCC samples. LASSO regression analysis was used for key gene selection and risk model construction for HCC overall survival. The model performance was estimated and validated. The key genes were validated for their heterogeneities in HCC cell lines with quantitative real-time PCR and at single-cell level. Their dysregulations were investigated at protein level. Their correlations with HCC response to anti-cancer drugs were estimated in HCC cell lines.

**Results:**

We identified three LSGs-based HCC subtypes with different prognosis, tumor stemness, and aging level. The C1 subtype with low LSGs score and high immune score presented a poor survival, while the C2 subtype with high LSGs score and immune score indicated an enduring survival. Although no significant survival difference between C2 and C3 HCCs was shown, the C2 HCCs presented higher immune score and stroma score. The HCC subtypes and their differences were confirmed in ICGC-HCC dataset. A five-gene prognostic signature for HCC survival was constructed. Its good performance was shown in both the training and validation datasets. The five genes presented significant heterogeneities in different HCC cell lines and hepatocyte subclusters. Their dysregulations were confirmed at protein level. Furthermore, their significant associations with HCC sensitivities to anti-cancer drugs were shown.

**Conclusions:**

LSGs-based HCC subtype classification and the five-gene risk model might provide useful clues not only for HCC stratification and risk prediction, but also for the development of more personalized therapies for effective HCC treatment.

**Supplementary Information:**

The online version contains supplementary material available at 10.1186/s12935-024-03242-3.

## Background

Liver cancer was reported to constitute 4.7% of the new cancer cases and 8.3% of the cancer deaths in 2020 [1]. Hepatocellular carcinoma (HCC) alone accounts for 75–85% of primary liver cancer cases and proves to be a serious economic and social burden [2, 3]. The poor prognosis of HCC is largely related to its late diagnosis at presentation. For instance, the 5-year survival rate of patients with early HCC after surgical resection is approximately 70%, while for the patients with advanced HCC, the 5-year survival rate is less than 20% [4, 5]. Treatment options for advanced HCC remain limited and ineffective, in part due to the high intra and inter tumoral heterogeneity associated with resistance and recurrence [6]. Thus, a better understanding of molecular classes and heterogeneity of HCC can aid in exploring molecular mechanisms of hepatocarcinogenesis and personalized therapeutic options.

Genome profiling of individual organs, tissues, and more recently, of single cells detect the molecular signatures of different biological samples, and reveal that the vast majority of cancer driver genes are mutated in a tissue-specific way [7]. Similar cancer hallmarks, such as proliferation and immune evasion, often demonstrate tissue specificity of the organization of oncogenic signaling pathways and are therefore followed by tissue-specific differences in treatment response and resistance [8, 9]. Tissue specificity in cancer may be partly originated from tissue-specific genes (TSGs) that are expressed at different levels across tissues, where both cell-intrinsic and cell-extrinsic factors play a role [10, 11].

Some TSGs, such as epidermal growth factor receptor (EGFR), carcinoembryonic antigen (CEA), and prostate-specific membrane antigen (PSM), are utilized as biomarker to assist diagnosis and prognosis of various types of tumors whose cells express these TSGs [12], whilst HCC has no molecular markers that have been incorporated into clinical management [13]. A variety of liver-specific genes (LSGs) were presented to be associated with HCC development and progression. For instance, RDH16 was shown to be downregulated in HCC and associated with HCC survival [14, 15]. Inverse association of SLC10A1 with HCC occurrence and progression were reported [16]. APOC1 was found to be decreased and negatively correlated with PD1/PD-L1 in HCC samples [17]. In our previous study, three LSGs including SPP2, UROC1, and SLC22A10 were found to be down-regulated in HCC and associated with prognosis of the patients [18]. However, systematic and high-throughput studies of LSGs in HCC are currently lacking.

In the past, there emerged simultaneous efforts to categorize HCC prognostically and therapeutically according to different molecular subclassifications, including patterns of mutational, genomic, histological, and clinical differences, though, no single consensus has been reached [19]. The efforts have made progress, but the task remains incomplete. We proposed that LSGs profiling of HCC would help to establish a subclassification system with molecular and prognostic features, and better stratify disease treatment by grouping patients into distinct molecular classes.

In the present study, we focused on LSGs to investigate their dysregulation and expression heterogeneity in HCC and explore their clinical significance. Based on LSGs expression, we identified and validated three HCC subtypes with different clinical and pathological features. With LSGs and HCC subtype-specific genes, a five-gene signature was constructed and the effectiveness in discriminating HCC survival status was validated. The expression heterogeneity of the five genes was visualized in a single cell RNA (scRNA)-seq dataset. Their dysregulations were confirmed at protein level. Furthermore, the five genes were also investigated for their associations with HCC treatment response to anti-cancer drugs. These results might provide new clues for HCC risk stratification, prognosis prediction, and personalized therapy.

## Materials and methods

### Data collection and processing

The RNA-seq data of HCC patients and their clinical features in TCGA (TCGA-LIHC, named TCGA-HCC dataset in this study, *n* = 371) and ICGC (LIRI-JP, named ICGC-HCC dataset in this study, *n* = 231) were downloaded. Htseq-count profiles were transcript per million (TPM) transformed for normalization. Single-cell transcriptomes data of 21 tissue samples (non-tumor liver, *n* = 8; primary tumors, *n* = 10; metastatic lymph node, *n* = 1; portal vein tumor thrombus, *n* = 2) from 10 HCC patients in GSE149614 were obtained from Gene Expression Omnibus (GEO). The proteomics data of 35 HCC tissues and their paired normal livers were retrieved from a previous study [20]. The clinical features of the patients were shown in Additional file 1: Table S1.

### LSGs-based HCC subtypes identification and their associations with HCC clinical and pathological features

The LSGs were obtained from tissue-specific gene expression and regulation (TiGER) database and Human protein atlas (HPA) and 138 common genes with higher expression in liver in the two databases were included. With the LSGs, consensus clustering (with ConsensusClusterPlus R package) was used for subtype identification of primary HCCs in TCGA-HCC dataset. The prognostic effects of HCC subtypes (clusters) on HCC overall survival (OS) and disease-free survival (DFS) were investigated through Kaplan-Meier survival analysis and multivariable Cox regression analysis. With “ggstatsplot” R package, the tumor stage and tumor grade proportions of different HCC subtypes were also compared.

### The associations of HCC subtypes with HCC stemness, aging, liver function, and immune response

The stem-cell signature in Bhattacharya study [21] (BHATTACHARYA EMBRYONIC STEM CELL.v7.5.1) from the Molecular Signatures Database (MSigDB) and a new aging-related gene set in Soul study [22] were retrieved. Based on the two gene sets and LSGs expressions, gene set variation analysis (GSVA) was applied to estimate the stemness score, aging score and LSGs score of the samples. The immune score, stromal score, and estimate score of HCC samples were estimated with “tidyestimate” R package. The stroma score was estimated based on the stroma gene expressions which could indicate the relative abundance of the stroma cell infiltration. The estimate score was the sum of the immune score and stroma score and it was used to infer tumor purity. As higher immune cell and stroma cell infiltration indicated fewer tumor cells, a higher estimate score indicated a lower tumor purity of the tumor samples. The infiltrations of 28 kinds of immune cells in different HCC samples were also evaluated with the cell markers retrieved from the cancer immunome atlas (TCIA, https://tcia.at/home). Wilcoxon test was used for comparisons of the scores and immune infiltrations between normal and HCC samples or between different HCC subtypes. The relations of LSGs score, stemness score, and aging score were evaluated with Spearman correlation analysis.

### Exploration of differentially expressed genes (DEGs) between the HCC subtypes and their potential functions

The gene expression profiles between different HCC subtypes were compared with “limma” R package. The genes with expressional difference of |log_2_(fold change)| (|logFC|) > 1 and adjusted *p* value < 0.05 was considered as significant DEGs. The DEGs with consistently higher or lower expression in each HCC subtype than the other two subtypes were considered as HCC subtype-specific DEGs. The subtype-specific DEGs were applied to gene ontology (GO) enrichment analysis with “clusterProfiler” package in R. With the Reactome pathways as the gene sets background, gene set enrichment analysis (GSEA) was also performed to explore the pathway activity differences between every two of the HCC subtypes. Then, the GSEA results were intersected to find HCC subtype-specific pathways.

### LSGs methylation and risk factor comparisons among HCC subtypes

To investigate the roles of epigenetic modifications, environmental factors, and viral hepatitis in shaping LSGs expressions and HCC subtypes, the methylations of the LSGs and risk factor compositions of the TCGA-HCC tumors were compared among different HCC subtypes. The alcohol assumption and viral infection status of the patients were obtained and the tumors were separated into different groups based on their risk factors. The associations of the LSGs expressions with different risk factors were also estimated through the comparisons among different risk factor groups (risk_group). Kruskal-Wallis test and Char-square test was used for comparisons and *p* < 0.05 was considered significant.

### Validation of LSGs-based HCC subtypes in internal and external datasets

For the validation of LSGs-based HCC subtypes, we used support vector machine (SVM) (https://CRAN.R-project.org/package=e1071) and nearest template prediction (NTP) [23] methods. The top subtype-specific DEGs (with a threshold of |logFC| >3) were used to classify the HCC subtypes in further analyses. With SVM algorithm, with the top subtype-specific DEGs, the discrimination model was constructed in 60% patients of TCGA-HCC dataset (training set, *n* = 221) and validated in other TCGA-HCC patients (internal validation set, 40%, *n* = 150) and ICGC-HCC dataset. In contrast, for NTP predication, according to the algorithm, only the higher expressed genes of the top subtype-specific DEGs were used as subtype-specific genes and the samples were predicated. The effectiveness of the classifiers was evaluated in TCGA-HCC dataset and then applied to ICGC-HCC dataset. With the HCC subtype classification in ICGC-HCC dataset, the prognostic effects of the subtypes, as well as their associations with HCC proliferation, liver function, and HCC microenvironment, were validated.

### The construction and validation of HCC subtype-related risk model for HCC prognosis

Through Cox regression analyses with “ezcox” R package, the LSGs and HCC subtype-specific genes were investigated for their age-, sex-, and stage-corrected prognostic effects on TCGA-HCC OS. The genes with prognostic effects independent of age, sex and stage were included for least absolute shrinkage and selection operator (LASSO) Cox regression analysis to select the key genes with close relations to HCC OS. The analysis was performed with “glmnet” R package and a 10-fold cross-validation was used to determine the optimal lambda (λ) for risk model construction. With the coefficients and the relative expressions of the key genes, the risk score of each patient could be calculated as follows:

$$\text{risk} \,\text{score}={\sum }_{i=1}^{n}Coef\left(i\right)*expression\left(i\right)$$

where *n* indicates the number of the key genes with significant prognostic effects, *expression(i)* is the relative expression level of the key genes, and *Coef(i)* represents the coefficient of the gene estimated through LASSO regression analysis. The accuracy of the risk model in predicating HCC OS status was evaluated in TCGA-HCC dataset and validated in ICGC-HCC dataset through receiver operating characteristic (ROC) analysis. The survival differences between high- and low-risk HCC patients were visualized through Kaplan-Meier survival analysis. To investigate the independent prognostic roles of the risk model, age, sex, tumor stage, liver fibrosis and the level of serum AFP were used as control variables and multi-variable Cox regression analysis was performed with “ezcox” R package. Furthermore, to investigate the associations of the risk model with HCC proliferation and immune check points, the Spearman correlations of risk score with the expressions of proliferation marker MKI67 and immune check points PD1 (PDCD1), PD-L1 (CD274) and CTLA4 were calculated.

### Cell culture and quantitative real-time PCR (RT-qPCR) analysis

The expressional heterogeneities of the key genes were investigated in HCC cell lines including Huh7 (well-differentiated), SNU449 (low-moderately differentiated), HCCLM3 (highly metastatic), and HepG2 (well-differentiated) through RT-qPCR analysis. Huh7 and HepG2 cell lines were obtained from the Chinese Academy of Sciences Cell Bank (CASCB, China). SNU449 and HCCLM3 cells were purchased from ATCC (United states) and Beyotime (Shanghai, China), respectively. Huh7 cells, HCCLM3 cells, and HepG2 cells were cultured in Dulbecco’s modified Eagle’s medium (BI, Israel) containing 10% fetal bovine serum (FBS) and 1% penicillin streptomycin (PS). SNU449 cells were grown in RPMI-1640 medium (BI, Israel), supplemented with 10% FBS (Beyotime, China) and 1% PS. The cells were placed in a cell culture incubator (Thermo Fisher, USA) containing 5% CO_2_ at 37 °C.

According to the manufacturer’s instructions, total RNA was isolated with TRIzol reagent (Invitrogen, USA). The extracted RNA of the genes was reverse transcribed into cDNA using a High-Capacity cDNA Reverse Transcription Kit (Thermo Fisher, USA). RT-qPCR analysis was performed using the SYBR Green Master Mix Kit (Thermo Fisher, USA). The primers for the genes were provided in Table 1. The 2^−ΔΔCt^ method was used for the calculation of relative expression level normalized by ß-tubulin. The experiments were performed in triplicate. T-test were used for the gene expression comparisons between different cell lines.

**Table 1:** The primer sequences of the genes

Genes	Forward primers (5′-3′)	Reverse primers (5’-3’)
ADH4	AGTTCGCATTCAGATCATTGCT	CTGGCCCAATACTTTCCACAA
PON1	CTGATTGCGCTCACCCTCTT	CGGAGAGCATTAAGTCGTGTTTG
PZP	GGAGAAGGACTTATTCCACTGTG	ATCTTGCGTAGGCCCCTTTAT
MMP10	TGCTCTGCCTATCCTCTGAGT	TCACATCCTTTTCGAGGTTGTAG
NR0B1	CTCACTAGCTCAAAGCAAACGC	GCGCTTGATTTGTGCTCGT
β-tubulin	CTGGACCGCATCTCTGTGTACT	GCCAAAAGGACCTGAGCGAACA

### Single-cell investigation of key gene expressions in HCC

For single-cell analysis, the cells with fewer (*n* ≤ 300) expressed genes and the genes expressed in fewer (*n* ≤ 3) cells were excluded. Doublets were removed with “DoubletFinder” R package. With “Seurat” R package, further quality control was performed. Only the cells with mitochondrial gene counts proportion < 20%, the ribosomal gene counts proportion > 3% were included for further analysis. To depict the cell type compositions of different tissues, cluster analysis of single-cell transcriptome was performed using “FindNeighbors” and “FindClusters” functions with dimensions of 15 and a resolution of 0.8. With the cell markers obtained from CellMarker (http://xteam.xbio.top/CellMarker/), the cells in different clusters were annotated into different cell types. The results were visualized by the uniform manifold approximation and projection (UMAP) method which projected the cells into two dimensions for visualization. The key genes in the risk model and the cell cluster marker genes were visualized for their expression in different cell types using “dotplot” function. To investigate the heterogeneities of the hepatocytes (normal hepatocytes or HCC tumor cells), the hepatocytes were extracted and subcluster analysis was performed. The proportions of different hepatocyte subclusters in different groups were calculated and presented with heatmap plot. To visualize the key gene expression profiles in different groups and subclusters, the subclusters were divided into different groups according to their proportions, with their highest proportion in the tissues as their tissue group. Then the differences of the key genes in different groups and subclusters of hepatocytes were visualized with “Vlnplot” function.

### Validation of the key gene dysregulations in HCC at protein level

The protein levels were compared between 35 pairs of HCC tissues and normal livers. Their associations with HCC proliferation were explored through their Spearman correlations with MKI67 expression. Their relations to HCC invasion were investigated through their expressional differences between the HCC tissues with and without microinvasion through paired Wilcoxon test or Chi-square test.

### Further exploration of the association of the key genes with HCC sensitivity to anti-cancer drugs

The key gene expressions in the cancer cell lines and the half maximal inhibitory concentrations (IC50s) of the anti-cancer drug were downloaded from Cancer Cell Line Encyclopedia (CCLE) database. After filtration, a total of 20 anti-cancer drugs and 19 HCC cell lines with the key gene expressions were included for analyses. The Spearman correlations of the anti-cancer drug IC50s with the key gene expressions were analyzed.

### The protein-drug and protein chemical interactions of the proteins coded by the key genes

Through NetworkAnalyst (https://www.networkanalyst.ca/), we investigated the protein-drug and protein-chemical interactions of the proteins coded by the key genes. The protein-drug interactions were obtained from the DrugBank database and the protein-chemical interaction data were collected from the Comparative Toxicogenomics Database (CTD).

### Statistical analysis

All the analyses in this study were performed with R 4.2.1. For Wilcoxon test, Chi-square test, Kruskal-Wallis test, spearman correlation analysis, Kaplan-Meier survival analysis, Cox regression analysis, and t-test, *p* < 0.05 was considered significant. For GO enrichment and GSEA, false discovery rate (FDR) < 0.05 was considered significant.

## Results

### LSGs-based HCC subtypes presented significant difference in clinical and pathological characters

**Fig. 1:**
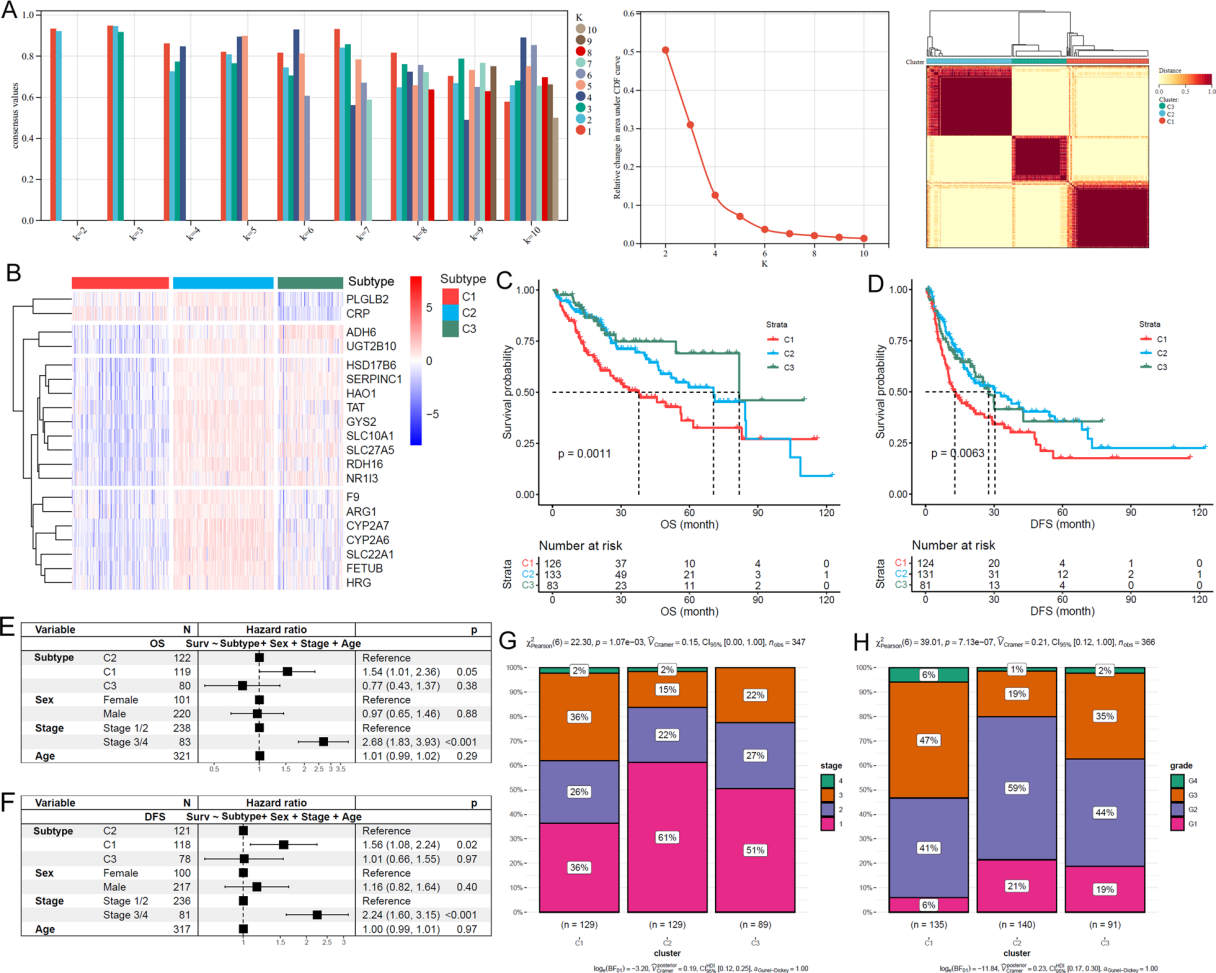
LSGs-based HCC subtypes and their differences in clinical characters. **A** Bases on LSGs expressions, HCCs were separated into three subtypes. **B** The expression profiles of 20 LSGs in TCGA-HCC. **C**, **D** Among the three subtypes, C1 HCCs presented shortest OS and DFS. **E**, **F** The unfavorable prognostic effects of C1 subtype on HCC OS and DFS were independent of age, sex, and tumor stage. **G**, **H** The comparisons of tumor stage and tumor grade among different HCC subtypes. Cluster analysis, Kaplan-Meier survival analysis with log-rank test, multivariable Cox regression analysis and Chi-square test were performed and *p* < 0.05 was considered statistically significant

Compared with normal liver tissues, 80.4% (111/138) of the LSGs were lower expressed in TCGA-HCC tissues (Additional file 1: Table S2). Based on the LSG expressions, three HCC subtypes (clusters: C1, C2 and C3) were identified (Fig. 1A). Through Kruskal-Wallis test, 96.4% (133/138) of the LSGs presented significant expressional differences among the three subtypes (Additional file 1: Table S3). A representative gene expression profile heatmap of 20 LSGs of the TCGA-HCC subtypes was shown in Fig. 1B, indicating the obvious heterogeneity of LSGs in HCC. For their prognostic differences, C1 presented the shortest OS and DFS (Fig. 1C, D). Notably, with sex, stage, and age as the control variables, the unfavorable prognostic effects of the C1 subtype on HCC OS and DFS also existed (Fig. 1E, F). In addition, tumor stage (Fig. 1G) and grade distribution (Fig. 1H) were significantly different among the subtypes of HCC and the proportions of late stage (stage III/IV) and high grade (Grade 3/4) HCCs were highest in C1, indicating the association of HCC subtypes with HCC progression and differentiation.

### Significant associations of HCC subtypes with HCC stemness, aging, liver function, and immune response

**Fig. 2:**
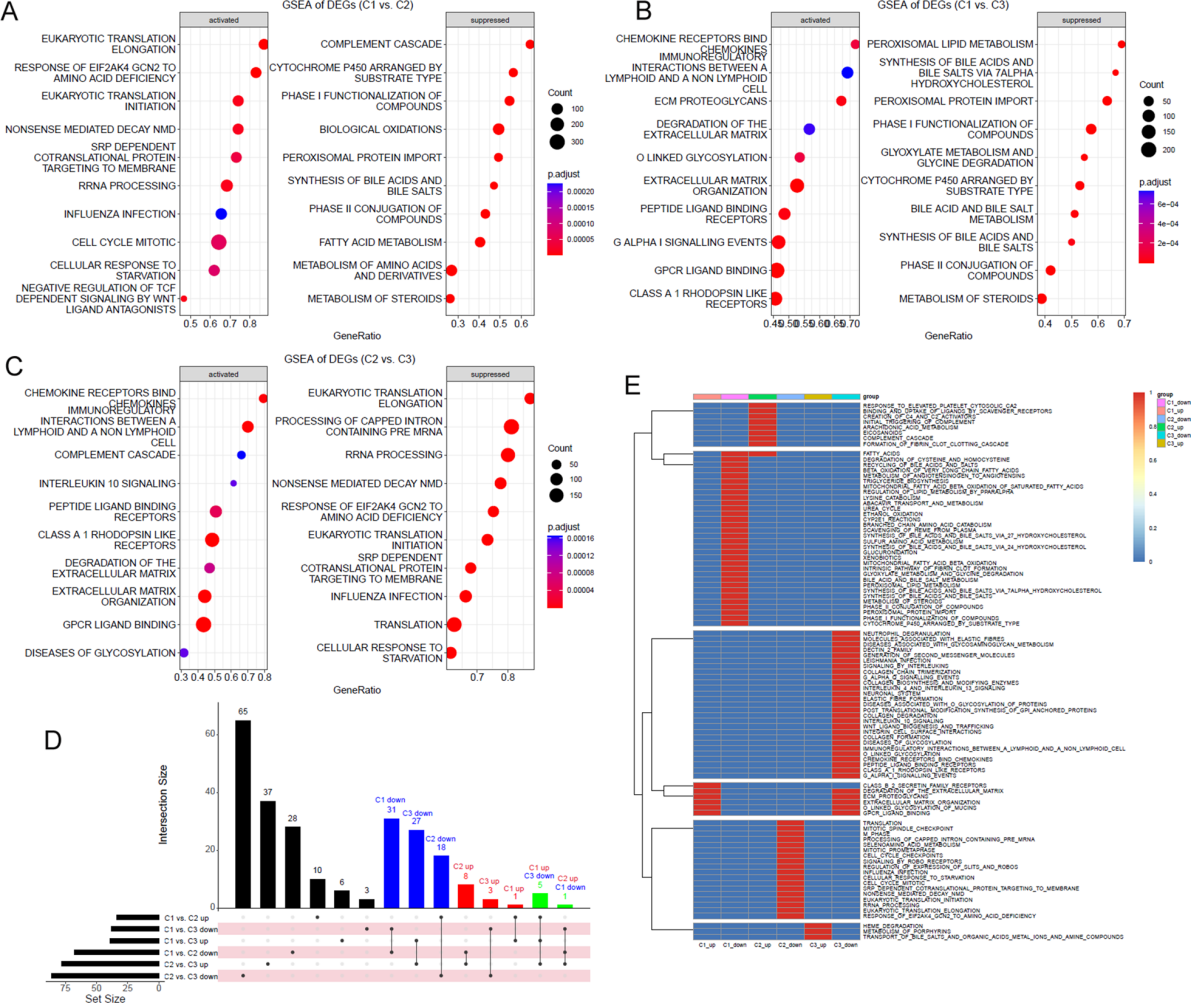
HCC subtype-specific pathways identified through GSEA analyses. **A**–**C** GSEA between C1 and C2, C1 and C3, and C2 and C3 subtypes, respectively. **D** The intersection of pathways among different HCC subtypes. **E** The HCC subtype-specific pathways comparing with other subtypes

As shown in Fig. 2, HCCs presented significantly lower LSGs score (Fig. 2A), higher stemness score (Fig. 2B), and lower aging score (Fig. 2C) than the normal liver controls, indicating the involvement of dysregulations of LSGs, stemness, and aging during HCC development. As the subtype identification was based on LSG expressions, it was not surprising to see the significant difference of LSGs score among different HCC subtypes and the C1 subtype with the poorest survival has the lowest LSGs score (Fig. 2D). The heterogeneity of stemness and aging level were also observed among the subtypes. C1 subtype presented the highest stemness score (Fig. 2E) and aging score (Fig. 2F), indicating the associations of HCC subtype with HCC stemness and aging. With regard to the relationship among the three parameters, as shown in Fig. 2G, H, LSGs score was significantly and negatively correlated with stemness and aging score in C3 HCC while no correlation was shown in C1 and C2 subtypes. However, all the three subtypes presented positive correlations between stemness score and aging score (Fig. 2I). The results indicated the complex interactions during HCC development. In addition, compared with C2 and C3 subtypes, C1 HCCs presented the highest MKI67 (Fig. 2J) and AFP expression (Fig. 2K). The highest ALB expression was shown in C2 HCCs, indicating the significant distinction of liver function among the different subtypes. The three HCC subtypes also presented differences in the microenvironment. As shown in Fig. 2M-O, compared with C1 and C2 HCCs, C3 had the lowest immune score, stroma score, and estimate score. As high estimate score indicated low tumor purity [24], the C3 were deduced to have the highest tumor purity among the three subtypes. The immune infiltration profiles in the subtypes were shown in Fig. 2P. Except for eosinophils and memory B cells, all of the other 26 kinds of immune cells were differentially infiltrated in the various HCC subtypes, with the lowest infiltration level in C3 HCCs of most of the immune cells (Additional file 2: Figure S1), consistent with the lowest immune score in C3.

### HCC subtype-associated DEGs and their potential functions

As shown in Additional file 2: Figure S2, 2030 genes were higher expressed while 772 genes were lower expressed in C1 than in C2. Compared with C3 HCCs, there were 3286 genes with higher expression while 715 genes with lower expression in C1 subtype. Additionally, 1329 genes were higher expressed while 545 genes were lower expressed in C2 than in C3. When the DEGs were intersected, there were 1812 C1-specific DEGs including 1445 highest expressed genes and 367 lowest expressed genes in C1 than in C2 and C3; 499 C2-specific DEGs (highest expressed: *n* = 290, lowest expressed: *n* = 209) and 1154 C3-specific DEGs (highest expressed: *n* = 244, lowest expressed: *n* = 910) were identified. The functions of the HCC subtype-specific DEGs were explored. Based on GO enrichment analysis, the C1-specific highest-expressed DEGs were significantly enriched in embryonic development related processes (Additional file 2: Figure S3A), while the C2-specific highest-expressed DEGs were associated with metabolic processes (Additional file 2: Figure S3B). For C3-specific highest-expressed DEGs, their associations with metabolic transport processes were obvious (Additional file 2: Figure S3C). Interestingly, similar to that of the C2-specific highest-expressed DEGs, the C1-specific lowest-expressed DEGs also presented significant associations with metabolic processes (Additional file 2: Figure S3D), which might partly account for the prognostic differences between the two subtypes. For the C2-specific lowest-expressed DEGs, their close relation to WNT signaling pathway was obvious (Additional file 2: Figure S3E). While for the C3-specific lowest-expressed DEGs, their associations with immune response and extracellular matrix organization were shown (Additional file 2: Figure S3F).

**Fig. 3:**
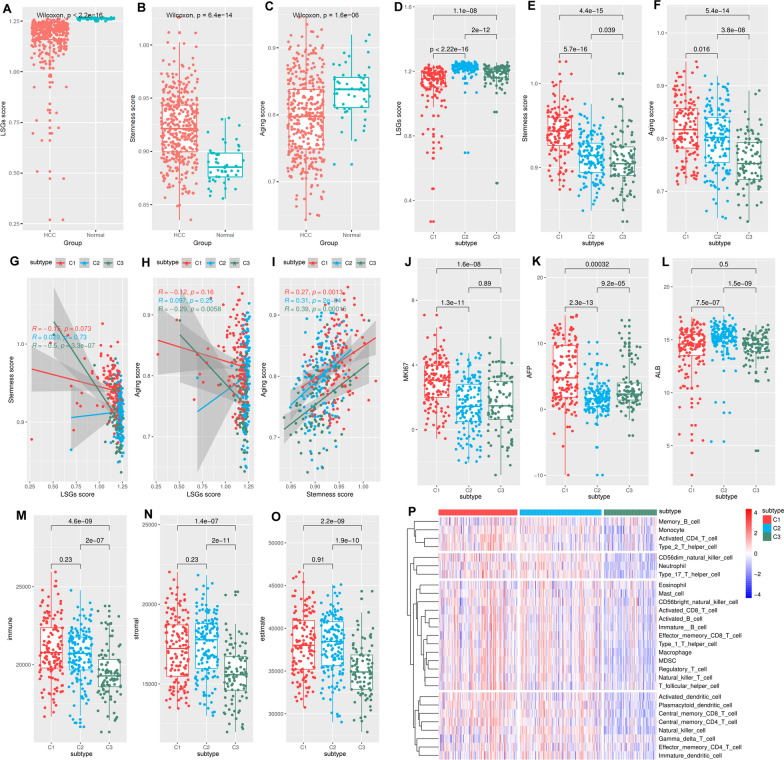
Different LSGs-based HCC subtypes presented differences in their stemness, aging and immune response levels. **A**–**C** Significant differences of LSGs score, stemness score, and aging score between HCC tumors and normal controls. **D**–**F** C1 HCCs presented lowest LSGs score while highest stemness score and aging score among the HCC subtypes. **G** The correlations between LSGs score and stemness score in HCC samples of different subtypes. **H** The correlations between LSGs score and aging score in HCC samples of different subtypes. **I** The significant positive correlation LSGs score and stemness score in HCC samples of different subtypes. **J**–**L** The expressional comparisons of MKI67, AFP, and ALB in HCC samples of different subtypes. **M**–**O** The comparisons of immune score, stromal score and tumor purity (negative correlated with estimate score) between HCC samples of different subtypes. **P** The heatmap of immune infiltrations in HCC samples of different subtypes. Wilcoxon test and Spearman correlation analyses were used and *p* < 0.05 was considered significant

Through GSEA analyses, the pathways with significant correlation with HCC subtypes were identified. The top10 pathways positively (activated) or negatively (suppressed) associated with the gene expression differences between either two of the HCC subtypes were shown in Fig. 3A-C. Based on intersection of the pathways (Fig. 3D), we found that the subtype-specific processes which were consistently activated (up-regulated) or suppressed (down-regulated) in one of the subtypes contrasted sharply with that in the others (Fig. 3E**)**. Among the three subtypes, the C1 subtype was positively correlated with six reactome pathways including o-linked glycosylation of mucins, GPCR ligand binding, extracellular matrix (ECM) organization, ECM proteoglycans, class B/2 secretin family receptors, and degradation of the ECM. Considering the crucial roles of ECM degradation in promoting cell migration, it indicated high potential of tumor metastasis in C1 HCCs. Various metabolic processes were negatively correlated with the C1 subtype, consistent with the GO terms “metabolic processes” enriched by the C1-specific lowest-expressed DEGs, indicating the weak liver function in C1 HCCs. As to the C2 subtype, its specific positive correlations with complement cascade, fatty acids, formation of fibrin clot clotting cascade, eicosanoids, arachidonic acid metabolism, and the binding and uptake ligands of scavenger receptors proposed its active metabolism and immune response. Most of the C2 subtype negatively-correlated pathways were about eukaryotic translation and cell cycle, suggesting its aberrant proliferation potential. For the C3 subtype, its positive relationships with porphyrins metabolism, substances (bile salts, organic acids metal ions and amine compounds), and heme degradation were consistent with the enriched GO terms “metabolic transport processes” of C3-specific highest-expressed DEGs. Moreover, similar to the GO terms enriched by C3-specific lowest-expressed DEGs, herein, the negative correlations of C3 subtype with extracellular matrix and immune response were also obvious.

### The associations of LSGs methylation and risk factor compositions with HCC subtypes

As shown in Additional file 1: Table S4, most (558/668, 83.5%) of the CpG sites of the LSGs presented significant differences of their methylation levels among the HCC subtypes. The top-50 CpG site with significant differences were shown in Additional file 2: Figure S4 and the representative CpG sites with significant difference were visualized in Additional file 2: Figure S5. It was obvious that the C1 subtype with the poorest survival had the highest methylation levels, consistent with the lowest LSGs score of the subtype. Whilst, the risk factor compositions among different HCC subtypes presented no significant difference (Additional file 2: Figure S6). In addition, among the 138 LSGs, only 21 genes (21/138, 15.2%) presented significant differences of different risk factors (Additional file 1: Table S5), indicating that the impact of the risk factors on LSGs-based HCC subtypes was relatively small.

### Validation of HCC subtypes in external dataset

**Fig. 4:**
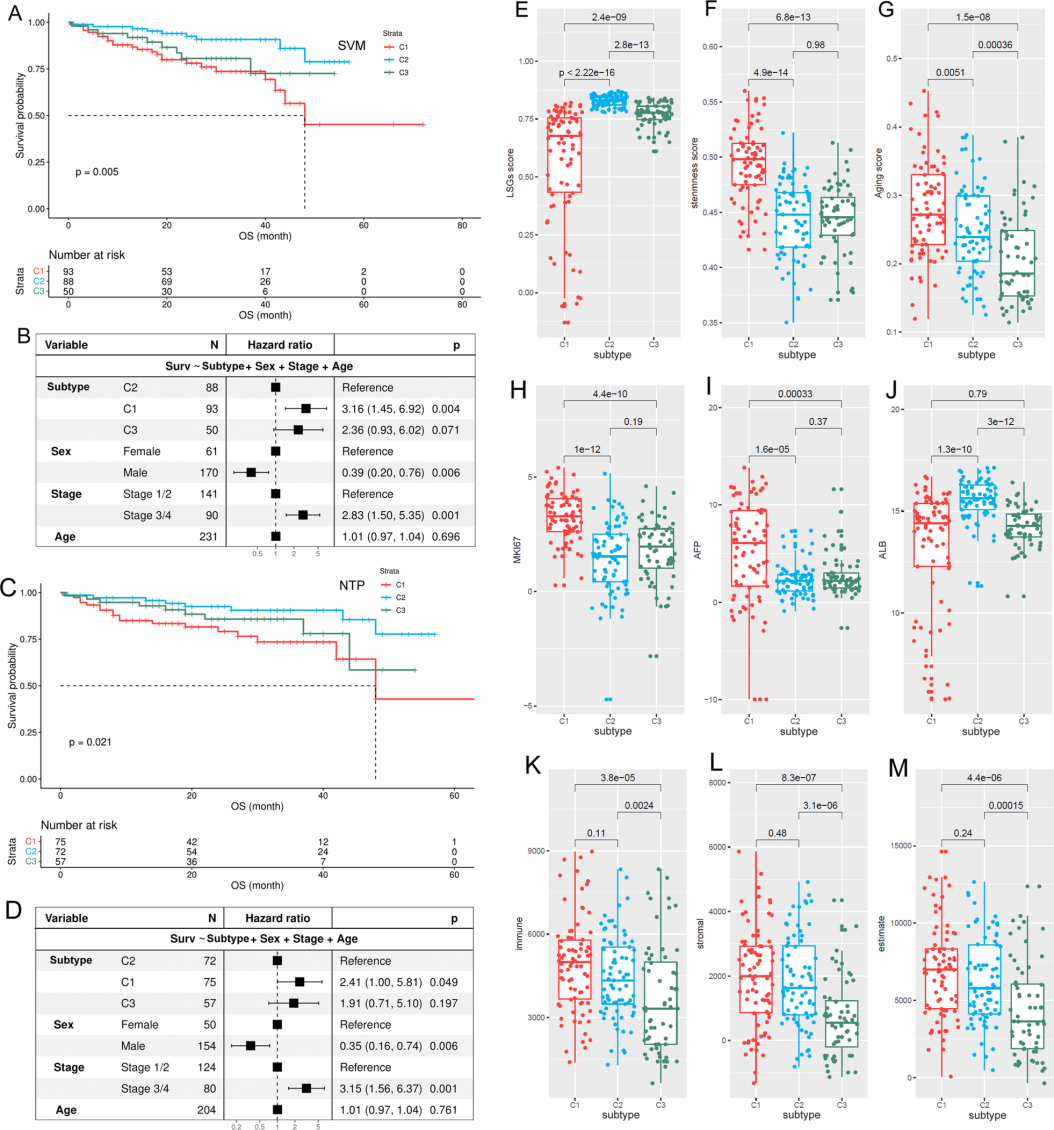
HCC subtype validation in ICGC-HCC dataset. **A**, **B** Prognostic effects of SVM-based HCC subtype on HCC OS. **C**, **D** Prognostic effects of NTP-based HCC subtype on HCC OS. **F**, **G** Comparisons of LSGs score, stemness score, and aging score in different ICGC-HCC subtypes. **H**–**J** Comparisons of LSGs score, stemness score, and aging score in different ICGC-HCC subtypes. **K**–**M** Comparisons of immune score, stromal score, and estimate score (tumor purity) in different ICGC-HCC subtypes

Among the subtype-specific DEGs, with the threshold of |log_2_FC| >3 and adjusted *p* < 0.05, the top HCC subtype-specific DEGs (*n* = 159) were identified (Additional file 1: Table S6). The top specific DEGs for C1, C2, and C3 subtype were 64 (highest: 44, lowest: 20), 28 (highest: 20, lowest: 8), and 70 (highest: 41, lowest:29), respectively. As some genes were highest expressed in one subtype while lowest expressed in another subtype, there were 159 unique genes of all. With these genes as variables, based on SVM analysis, a discriminant model was constructed in TCGA-HCC dataset. The SVM model could discriminate 97.3% of the training set samples and 84.7% of the internal validation set samples, with an overall accuracy of 92.1% (Additional file 1: Table S7). When the model was applied to ICGC-HCC dataset, 93, 88, and 50 of the primary HCC samples were classified to be C1, C2, and C3 subtype, respectively (Additional file 1: Table S8). Based on Kaplan-Meier analysis and multivariable Cox regression analysis, the poorest OS outcome of C1 subtype in ICGC-HCC dataset was also shown (Fig. 4A) and the independent prognostic effects of C1 subtype was indicated (Fig. 4B).

Considering the possible overfitting potential of SVM method, we also used NTP method for HCC subtype validation. Taking the top subtype-specific highest expressed DEGs as the subtype-specific genes, the TCGA-HCC samples could be classified with an accuracy of 82.2% (Additional file 1: Table S9). When the threshold was set to FDR < 0.05, 276 of 318 (accuracy: 86.8%) TCGA-HCC samples could be correctly discriminated. With FDR < 0.1, the accuracy was 85.5% (284 of 332). Herein, for the consideration of the accuracy and the sample size, we selected FDR < 0.1 as the threshold to evaluate the HCC subtypes in ICGC-HCC dataset. As shown in Additional file 1: Table S10, 204 of 231 (88.3%) ICGC-HCC samples met the criterion of FDR < 0.1 that 75, 72, and 57 HCC samples were classified into the C1, C2, and C3 subtypes, respectively. Through survival analysis (Fig. 4C, D), HCC patients of the HCC subtypes presented significant differences in their OS status and the C1 subtype tended to have the poorest outcome. The results were similar to that in Fig. 4A and B, in confirmation of the unfavorable effects of the C1 subtype in TCGA-HCC dataset. In addition, consistent with the results in TCGA-HCC dataset, the C1 ICGC-HCCs also presented the lowest LSGs score (Fig. 4E) and the highest stemness score (Fig. 4F) and aging score (Fig. 4G) among the three subtypes. In addition, MKI67 (Fig. 4H), AFP (Fig. 4I), and ALB (Fig. 4J) level, as well as the immune score (Fig. 4K), the stromal score (Fig. 4L), and the tumor purity (estimate score) (Fig. 4M), also showed the similar results to those in TCGA-HCC dataset.

### The HCC subtype-related risk model for HCC prognosis

**Fig. 5:**
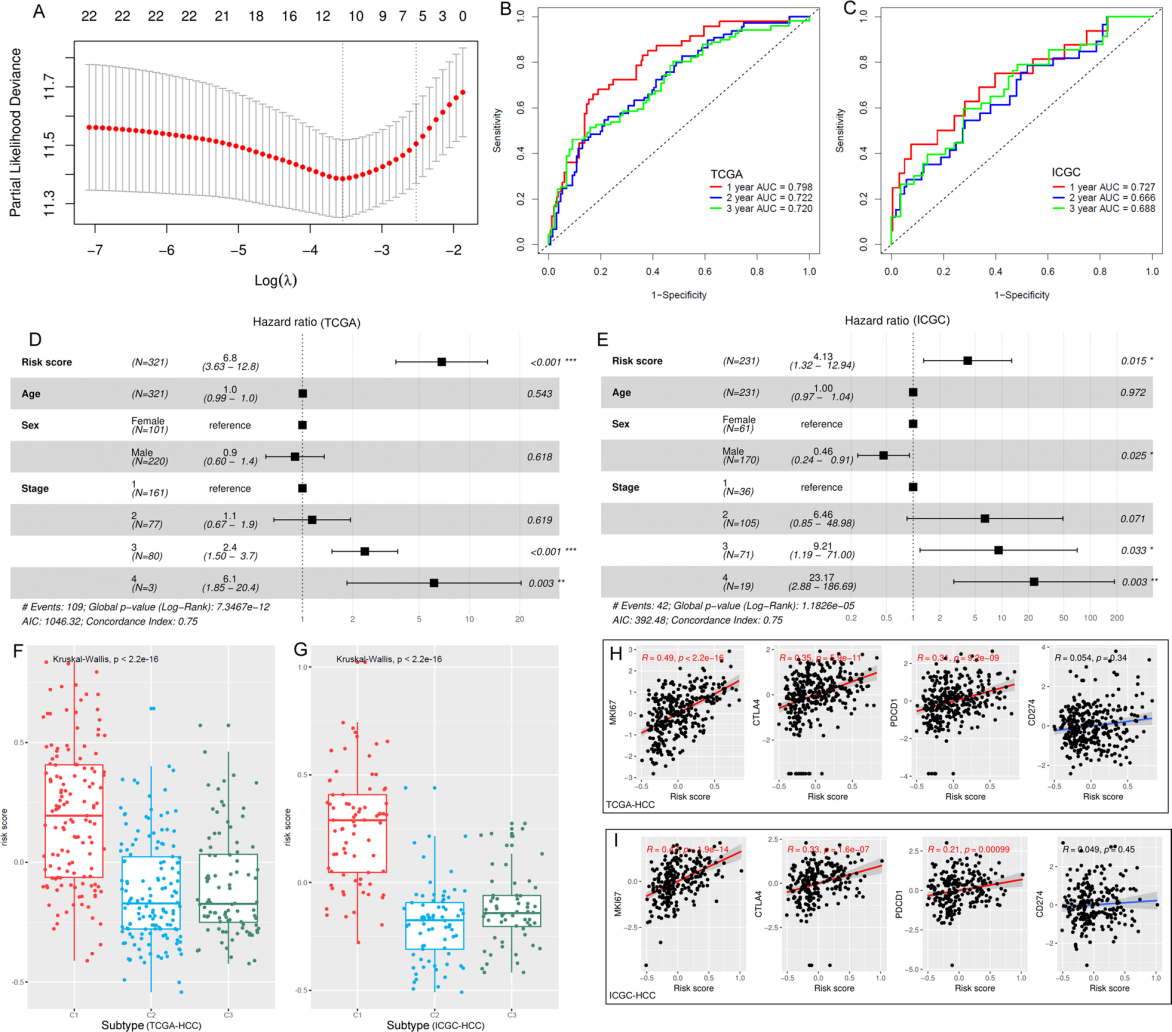
HCC subtype-related risk model and its correlations with HCC pathological features. **A** The tuning parameter lambda (λ) selection in LASSO Cox regression analysis using 10-fold cross-validation. **B**, **C** The risk model could effectively discriminate the 1-year, 2-year, and 3-year OS status of HCC patients in the training set (TCGA-HCC) and validation dataset (ICGC-HCC). **D**, **E** The independent prognostic effects of risk score in TCGA-HCC and ICGC-HCC datasets. **F**, **G** C1-HCCs presented the highest risk scores among all the HCCs in both TCGA-HCC and ICGC-HCC dataset. **H**, **I** The correlations of risk score with MKI67, CTLA4, PD1, and PD-L1 expressions in TCGA-HCC and ICGC-HCC samples

The LSGs and the HCC subtype-specific genes were investigated for their age, sex, and stage-corrected prognostic effects. Based on multi-variable Cox regression analyses, 64 genes were shown to be the prognostic indicators for TCGA-HCC OS, 22 of which were with *p* < 0.01 (Additional file 1: Table S11). As shown in Fig. 5A, the 22 genes were applied to LASSO Cox regression analysis and five (ADH4, PON1, PZP, MMP10, and NR0B1) of them were identified to be independent prognostic factors and were included in the risk model for HCC OS prediction. The risk model was as below:

$${\text{Risk score = - 0}}{\text{.046*ADH4exp - 0}}{\text{.062*PON1exp - 0}}{\text{.043*PZPexp + 0}}{\text{.091*MMP10exp + 0}}{\text{.192*NR0B1exp}}$$

Based on ROC analysis, the risk model could discriminate 1-year, 2-year, and 3-year OS of TCGA-HCC patients with AUCs of 0.798, 0.722, and 0720, respectively (Fig. 5B). With the AUCs of 0.727, 0.666, and 0.688 in the discrimination of 1-year, 2-year, and 3-year OS of ICGC-HCC patients (Fig. 5C), the effectiveness of the risk model was confirmed. In Additional file 2: Figure S7, the higher risk score was shown to be significantly associated with the shorter survival in both TCGA-HCC and ICGC-HCC patients. Taking age, sex, and stage as control variables, the prognostic effect of the risk model was also valid (Fig. 5D-E), indicating the independent role of the risk score in predicting HCC OS. Additionally, when incorporating liver fibrosis, serum AFP, and the risk score into the survival analysis, only risk score was presented to be an independent prognostic indicator for HCC OS, while no significance of liver fibrosis and serum AFP was shown (Additional file 2: Figure S8). In Fig. 5F-G, among the three subtypes, C1 HCCs presented the highest risk scores in both TCGA-HCC and ICGC-HCC datasets. The expressional differences of the five genes among the HCC subtypes were evident and the heterogeneity was visualized in Additional file 2: Figure S9. Furthermore, the associations of the risk model with HCC proliferation and the immune check points were obvious. As shown in Fig. 5H-I, the HCC risk scores presented significant positive correlations with  the proliferation marker MKI67, and two immune check point genes PD1 (PDCD1) and CTLA4. Nevertheless, no significant correlation between the risk score and PD-L1 (CD274) expression was discovered in the HCC tissues.

### Expressional heterogeneity of the key genes in HCC cell lines

**Fig. 6:**
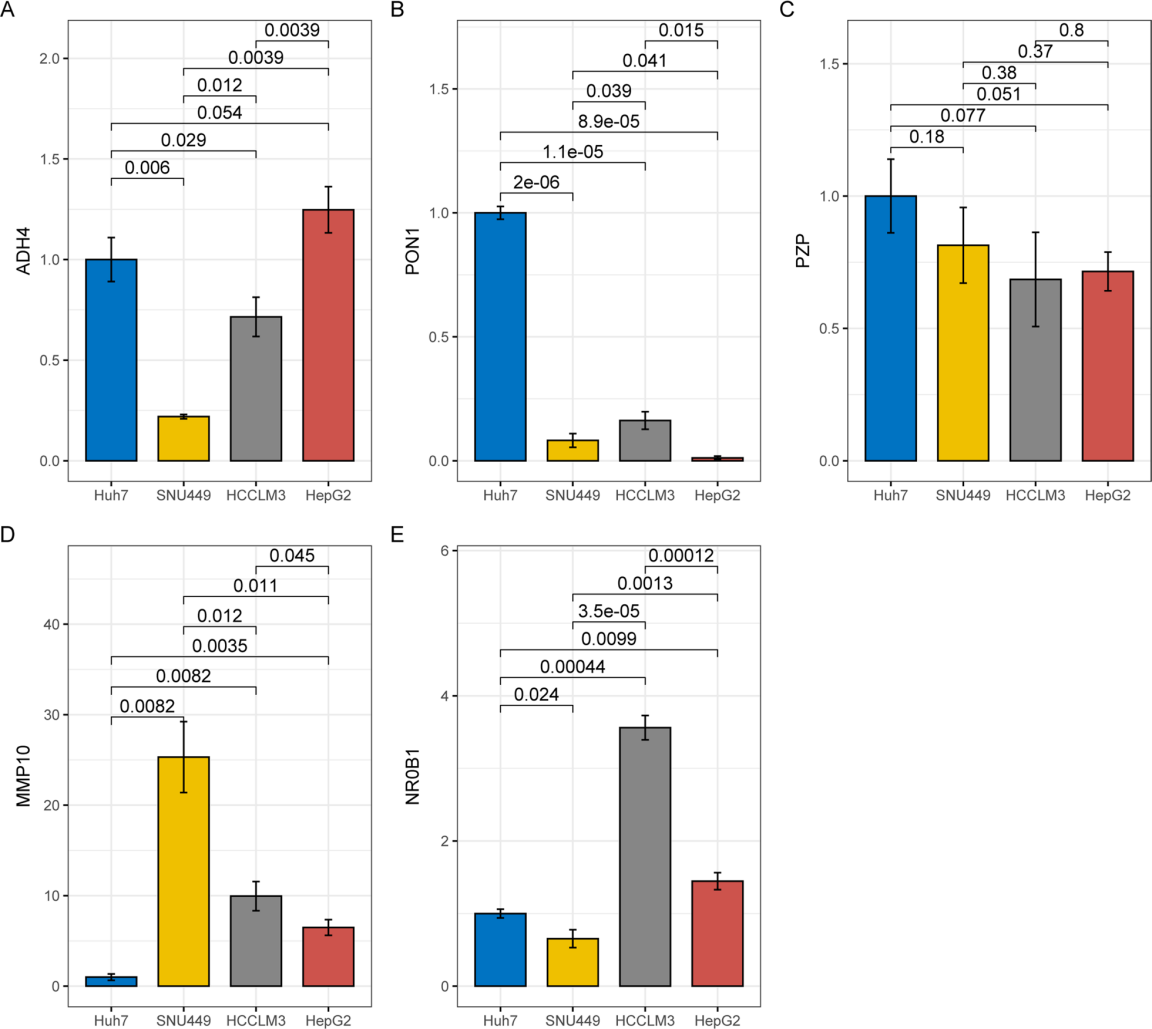
RT-qPCR analysis of the key gene expressions in different HCC cell lines. **A** Lower expression of ADH4 in SNU449 cells and HCCLM3 cells than Huh7 cells and HepG2 cells. **B** Significant higher expression of PON1 in Huh7 cells than SNU449 cells, HCCLM3 cells, and HepG2 cells. **C** Expressional comparisons of PZP in Huh7 cells, SNU449 cells, HCCLM3 cells, and HepG2 cells. **D** Expressional comparisons of MMP10 in Huh7 cells, SNU449 cells, HCCLM3 cells, and HepG2 cells. **E** Expressional comparisons of NR0B1 in Huh7 cells, SNU449 cells, HCCLM3 cells, and HepG2 cells

As shown in Fig. 6, significant differences of the key gene expressions among the HCC cell lines were shown. Both SNU449 cells and HCCLM3 cells presented a lower expression of ADH4 (Fig. 6A) than the well-differentiated Huh7 cells and HepG2 cells, indicating the positive associations of ADH4 with HCC differentiation. For PON1, its expressional differences among the four cell lines were obvious and Huh7 cells presented the highest expression of PON1 (Fig. 6B). Although no significant difference of PZP expression was shown among the cell lines, a tendency of lower level of PZP was indicated in HCCLM3 and HepG2 than in Huh7 cells (Fig. 6C). With regard to MMP10 expression (Fig. 6D), among the four cell lines, the lowest level was shown in Huh7 cells. Noticeably, SNU449 cells with low-moderate differentiation and HCCLM cells with high metastasis presented higher expression of MMP10 than the well-differentiated Huh7 cells and HepG2 cells, indicating the association of MMP10 with HCC metastasis and malignancy. For NR0B1, its expressional differences were indicated in the four cell lines, with the highest expression in highly metastatic HCCLM3 cells. The results represented the expressional heterogeneity of the key genes among different HCC cell lines, indicating the variation of the key gene expressions might be associated with HCC progression.

### Single-cell investigation of key gene expressions in HCC

**Fig. 7:**
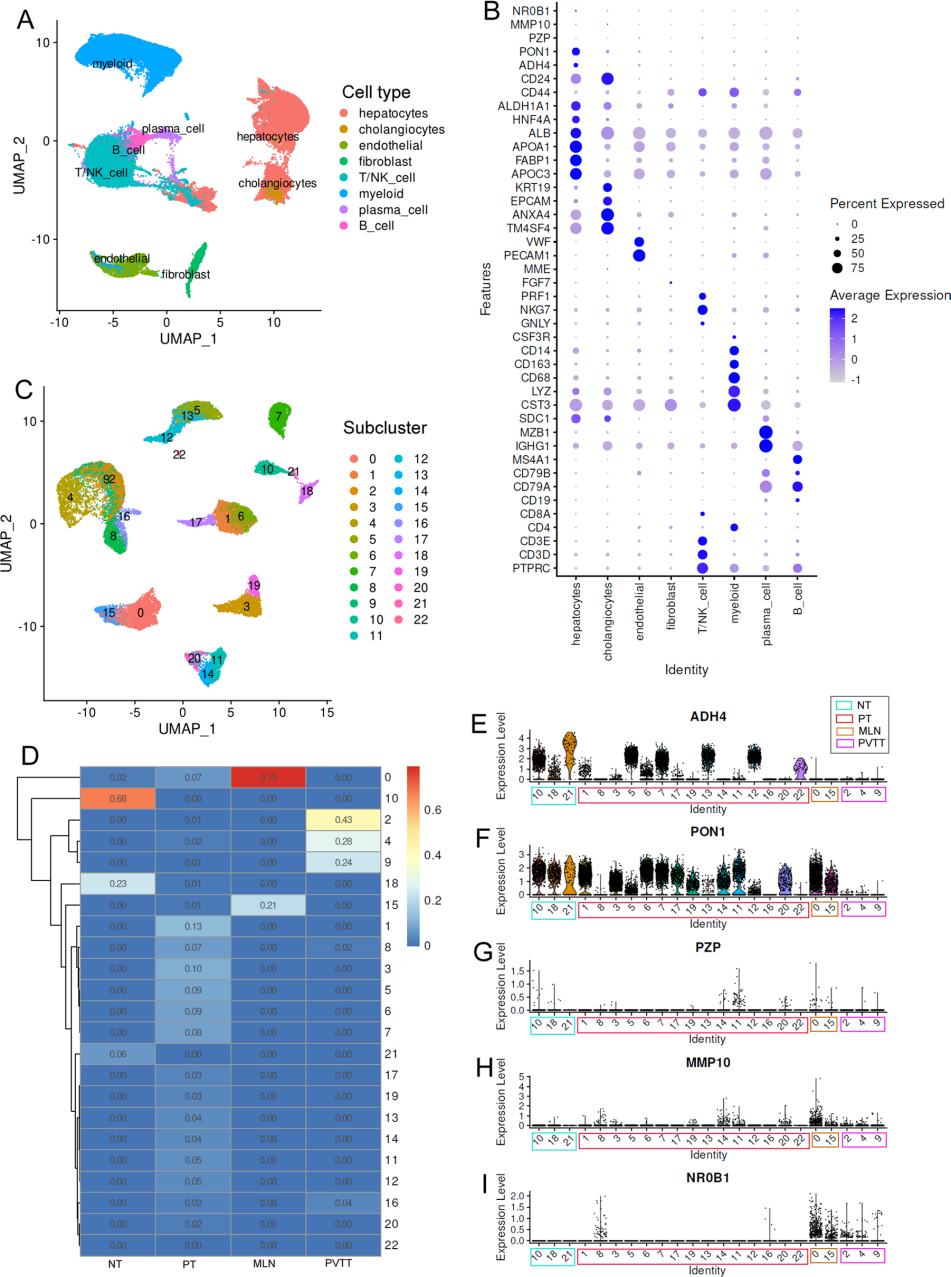
The expressional heterogeneity of the five key genes in hepatocyte-originated cells. **A** Cell types of liver and HCC tissues at single-cell level. **B** Marker gene and key gene expression profiles in different cell types. **C** Subclusters of hepatocytes in liver tissues and HCC samples. **D** The proportions of different hepatocyte subclusters in liver and HCC samples. **E**–**I** The expressional profiles of the five key genes in different hepatocyte subclusters. NT, non-tumor liver; PT, primary tumor; MLN, metastatic lymph nodes; PVTT, portal vein tumor thrombus

A total of 67,970 cells originated from tumor and normal tissue were classified into 47 clusters (Additional file 2: Figure S10A). Based on the gene expression profiles of different clusters (Additional file 2: Figure S10B), the single cells were grouped into eight major cell types (Fig. 7A). The expression profiles of the representative makers of the cell types and the key genes in the risk model were shown in Fig. 7B. Notably, ADH4 and PON1 were prominently expressed in hepatocyte-originated cells (named hepatocytes in this study) while the other three key genes were generally low expressed in all of the cell types. The significant expressional differences of the five genes were visualized in Additional file 2: Figure S11 and four of them presented the highest positive expression rate in hepatocytes. To investigate the expressional heterogeneity of the key genes, the hepatocytes were focused for further study. Adopting the UMAP method, the hepatocytes were divided into 23 subclusters (subcluster 0–22) (Fig. 7C) and their proportions in different tissues were significantly different (Additional file 1: Table S12). As shown in Fig. 7D, in contrast to the compositions (hepatocyte subclusters) of normal liver tissues (three major subclusters and one minor subcluster), metastatic lymph nodes (two major subclusters), and portal vein tumor thrombus (three major subclusters and one minor subcluster), the hepatocytes in the primary tumors indicated the highest heterogeneity of the hepatocytes.

To visualize the key gene expression profiles in different tissues and subclusters, the 23 subclusters were divided into different groups according to their proportions, with their highest proportion in the tissues as their tissue group. The expressional profiles of ADH4, PON1, PZP, MMP10 and NR0B1were presented in Fig. 7E-I. ADH4 was commonly expressed in the three subclusters of normal livers while it was rarely expressed in some subclusters of primary tumors and all the subclusters in metastatic lymph nodes and portal vein tumor thrombus (Fig. 7E). For PON1, in contrast of its positive expression in hepatocyte subclusters of normal livers, metastatic lymph nodes, and primary tumors, its negative expression was shown in portal vein tumor thrombus (Fig. 7F). With regard to PZP (Fig. 7G), its positive expression was shown in only a small part of the subclusters while negative in the others. For MMP10 (Fig. 7H) and NR0B1 (Fig. 7I), their positive expression was only shown in tumor tissues, especially in the metastatic lymph nodes and portal vein tumor thrombus, indicating their associations with HCC metastasis. Furthermore, as shown in Additional file 2: Figure S12, all the five genes presented significant differences in the hepatocyte subclusters of the primary tumors. The other tissues also presented expressional heterogeneities of the genes (normal livers: ADH4 and PON1; metastatic lymph nodes and portal vein tumor thrombus: PON1 and NR0B1) in their hepatocyte subclusters. The expressional heterogeneities of the genes might, to some extent, account for the outcome distinction of the HCC patients.

### Dysregulations of the key genes at protein level

**Fig. 8:**
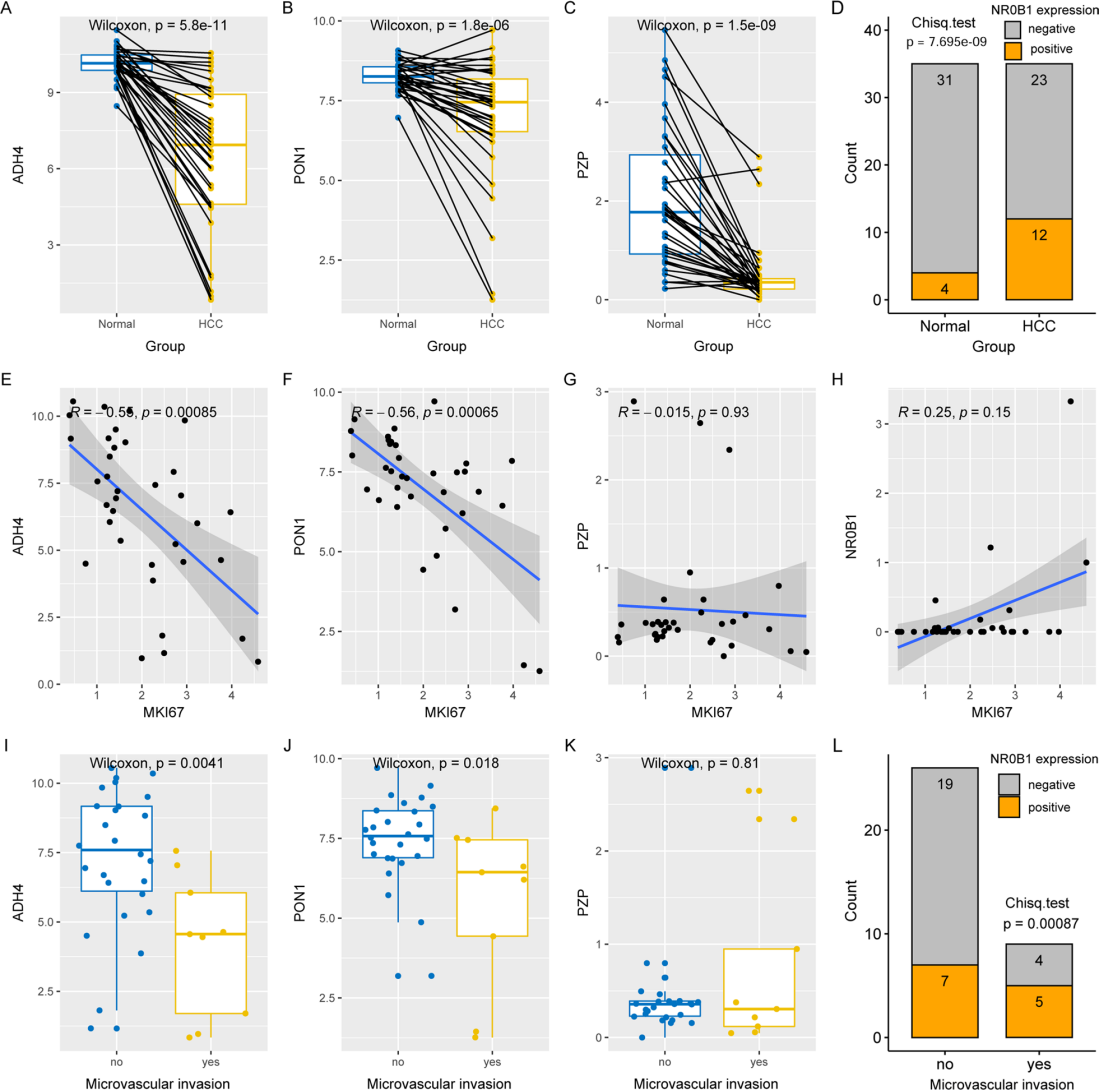
Dysregulations of ADH4, PON1, PZP, and NR0B1 in HCC at protein level. **A–C** ADH4, PON1, PZP were lower expressed in HCC tissues than their paired normal liver controls. **D** NR0B1 positive expression was higher in HCC tissues than their paired normal liver controls. **E**–**G** Spearman correlations of ADH4, PON1, PZP, and NR0B1 with MKI67 expression in HCC samples. **I–L** Comparisons of ADH4, PON1, PZP, and NR0B1 expressions between HCC samples with and without microvascular invasion. Wilcoxon test, Spearman correlation analysis, and Char-square test were used and *p* < 0.05 was considered significant

Compared with the paired normal tissues, ADH4, PON1, and PZP were significantly down-regulated in early-stage HCCs (Fig. 8A–C). In contrast, NR0B1 presented higher positive expressions in HCC tissues than in the normal controls (Fig. 8D). As shown in Fig. 8E, F, significant negative correlations of ADH4 and PON1 with MKI67 expression was obvious, indicating their negative association with HCC proliferation. However, no significant correlation of PZP and NR0B1 expressions with MKI67 was presented (Fig. 8G, H). As to their associations with HCC invasion, ADH4 and PON1 appeared to be negatively correlated with HCC microvascular invasion (Fig. 8I, J). In contrast, in the HCC tissues concerning microvascular invasion, high proportion of positive NR0B1 expression was obvious, indicating its positive association with HCC invasion (Fig. 8L). However, no significant correlation of PZP expression with HCC microvascular invasion was shown (Fig. 8K). For MMP10, at protein level, its contribution to HCC tumorigenesis and its elevated expression at HCC initiation has been reported in previous studies [25, 26].

### The associations of ADH4, PON1, PZP, MMP10, and NR0B1 with anti-tumor drug sensitivities of HCC cell lines

**Fig. 9:**
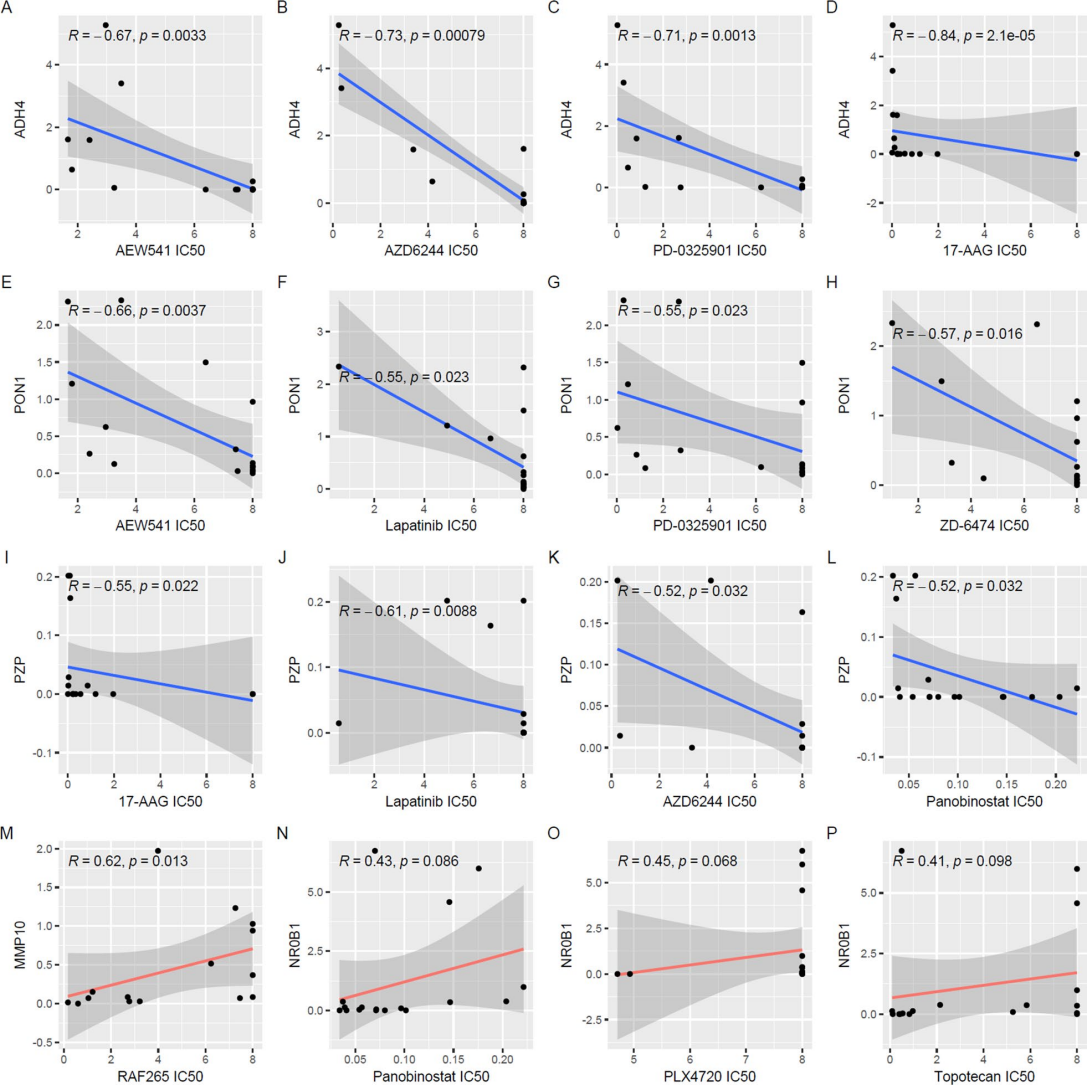
Correlations of the key gene expressions with anti-cancer drug sensitivity in HCC cell lines. **A**–**D** Significant negative correlation of ADH4 expression with IC50s of anticancer drugs in HCC cell lines. **E**–**H** Significant negative correlation of PON1 expression with IC50s of anticancer drugs in HCC cell lines. **I**–**L** Significant negative correlation of PZP expression with IC50s of anticancer drugs in HCC cell lines. **M** Significant positive correlation of PON1 expression with IC50 of RAF265 in HCC cell lines. **N**–**P** Positive correlation trend of NR0B1 expression with IC50 values of PLX4720, Panobinostat, and Topotecan in HCC cell lines. IC50s, half maximal inhibitory concentrations. Spearman correlation analysis was used and *p* < 0.05 was considered as statistically significant

As shown in Additional file 1: Table S13, ADH4 expression was found to be negatively correlated with the IC50 of 17-AAG, AZD6244, PD-0325901, AEW541, TAE684, Panobinostat, and RAF265 in HCC cell lines, indicating the positive association with HCC sensitivities to these anticancer drugs. Additionally, the negative correlation of PON1 with the IC50 of AEW541, ZD-6474, Lapatinib, and PD-0325901, and the negative association of PZP expression with the IC50 of Lapatinib, 17-AAG, Panobinostat, and AZD6244 in HCC were presented. For MMP10, its significant positive correlation with the IC50 of RAF265 in HCC were obvious. These representative significant correlations were visualized in Fig. 9A–M. Although none of the 24 anticancer drugs IC50s was shown to be statistically correlated with NR0B1 expression in HCC, there was a tendency of positive correlation (0.05 < *p* < 0.1) between NR0B1 expression and the IC50s of PLX4720, Panobinostat, and Topotecan (Fig. 9N–P).

### The protein-drug and protein chemical interactions of ADH4, PON1, PZP, MMP10, and NR0B1

**Fig. 10:**
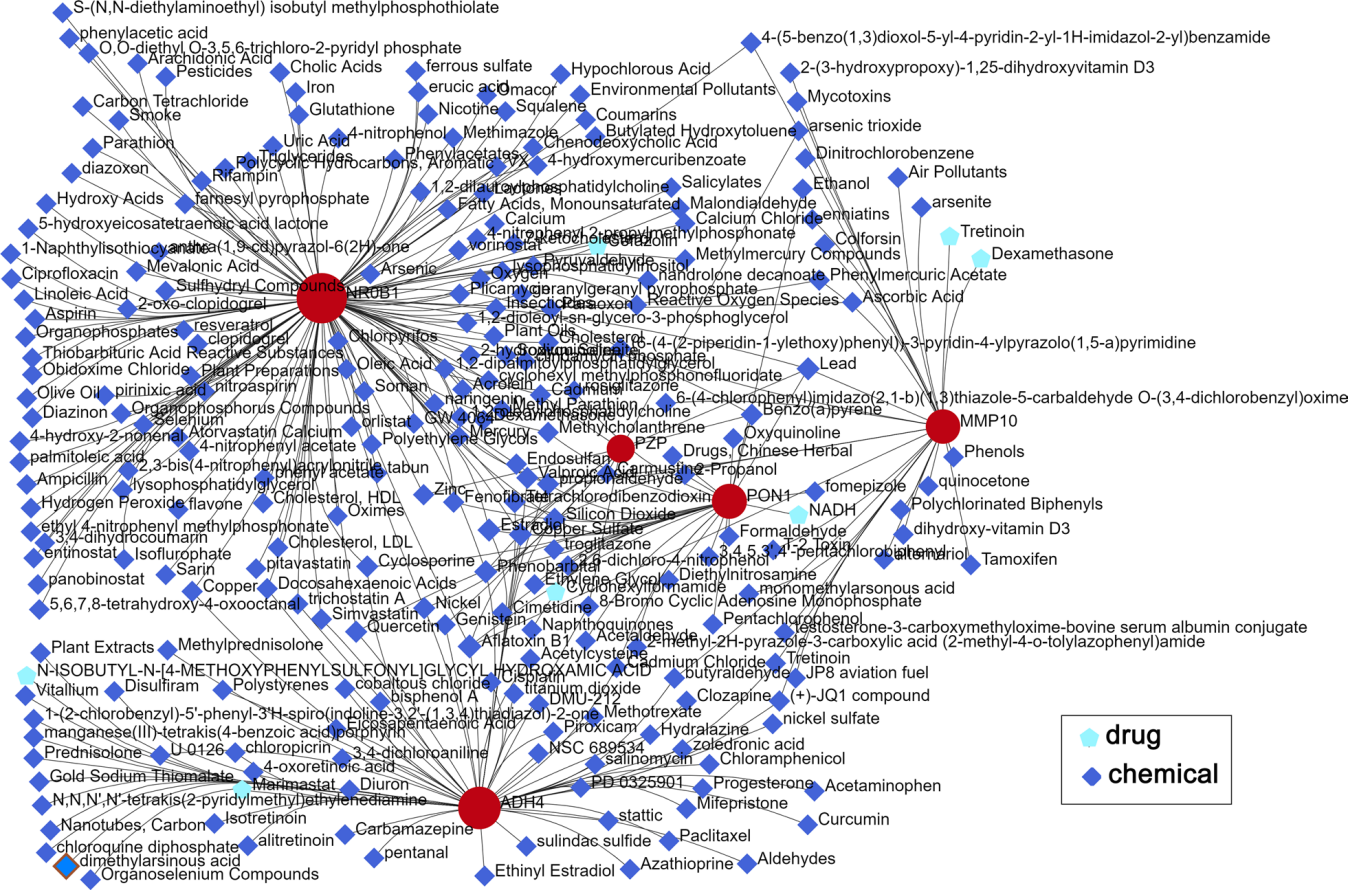
The protein-drug and protein chemical interactions of ADH4, PON1, PZP, MMP10, and NR0B1

As shown in Fig. 10, seven protein-drug interactions and 288 protein-chemical interactions of the five proteins were obtained. These drugs and chemicals might provide new clues and hints for the treatment and theraputic mechanism research of HCC.

## Discussion

HCC has a high degree of etiological and biological heterogeneity, resulting in distinct treatment responses. Considering the close relationship of LSGs with liver biofunctions [27, 28], systematic study of LSGs heterogeneity might provide novel clues for more personalized therapies in HCC. In the present study, we identified three LSGs-based HCC subtypes, among which the C1 subtype with the lowest LSGs score was highlighted to have the shortest OS and DFS and the highest stemness score, aging score, MKI67 expression, and AFP expression. In contrast to the significant differences in OS and DFS, the immune score, stroma score, and tumor purity were similar between C1 subtype and C2 subtype. Although C2 and C3 subtypes presented similar survival, their microenvironment response was significantly different. We also identified HCC-subtype specific genes and confirmed the subtype classification and the disparities among the three subtypes in external datasets. The expressional heterogeneities of the key genes were confirmed in HCC cell lines and hepatocyte subclusters at single-cell level. The findings demonstrated HCC heterogeneity in LSGs expressions and suggested the necessity of personalized treatment for different HCC subtypes.

Immunoreactivity differences between cancer subtypes could lead to distinct response to immunotherapies [29, 30]. C1 and C2 subtypes with higher immune score and immune cell infiltrations, might be more fit for immune checkpoint inhibitors (ICIs) therapy than C3. In addition, the higher expression of AFP and MKI67 in C1 HCC might be an indicator for the treatment with anti-proliferation drugs and AFP vaccine. Notably, the GSEA results and functional enrichment showed lower metabolic activities in the C1 HCCs than the other two subtypes, indicating their poorer liver function, which might affect treatment tolerance and efficiency. In contrast, C3 HCCs with high LSGs score and low immune reaction might be more suitable for chemotherapy or combination therapy. In previous studies, immune infiltrations were considered to be in “hot” tumors that were reported to have better survival [31, 32]. Herein, we found that high immune response with low LSGs score (C1 subtype) was related to poor prognosis, while high immune score with high LSGs score (C2 subtype) was an indicator for good survival. The inconsistent association of immune reaction with HCC survival was also reported in Shimada’s study [33]. These results suggested that more indicators were needed in combination with immune response for HCC prognosis predication and treatment option, and LSGs-based HCC classification provided an effective tool for HCC stratification.

Stemness and aging were also reported to be associated with HCC development and progression [34–37]. In our study, higher stemness score with lower aging score was shown in HCC than in normal livers, consistent with previous studies [38, 39]. Among the HCC subtypes, stemness score and aging score exhibited the highest level in C1 subtype with the poorest survival. The heterogeneity of HCC subtypes was also presented in the correlation of stemness score and aging score with LSGs score. Stemness score and aging score was significantly and negatively correlated with LSGs score in C3 HCC, while not in the other two subtypes, indicating distinctive involvement of LSGs in the development of C3 subtype HCC. Doxorubicin-induced senescence was reported to promote the stemness and tumorigenicity of HCC cells [40]. We also found the significant and positive correlation between stemness score and aging score in all the three subtypes, indicating their potential molecular synergy in HCC.

Through systemic analyses, we constructed an effective five-gene signature for HCC survival prediction. Three LSGs (ADH4, PON1, and PZP) and two C1-specfic genes (with highest expressions in C1 HCC: NR0B1 and MMP10) were shown to be independent prognostic factors for HCC and were included in the risk model. ADH4 coded an alcohol dehydrogenase enzyme, which was important for normal liver biofunction. ADH4 knockout mice were susceptible to vitamin A deficiency during gestation and had lower fertility survival rate [41]. PON1 coded a high-density lipoprotein-associated esterase, which had anti-oxidant and anti-inflammatory properties [42]. PON1 dysregulation was associated with a variety of cancers including breast cancer [43, 44], pancreatic cancer [45], endometrial Cancer [46], and colorectal cancer [47]. PZP was found as an anti-tumor gene with aberrant DNA methylation in many cancers [48–51]. NR0B1, an atypical orphan nuclear receptor, was implicated in embryonic stem cells and cancer biology [52]. MMP10 was a member of stromelysins, which was overexpressed and played a tumor-promotive role in oral cancer [53], ovarian cancer [54], pancreatic ductal adenocarcinoma [55], and colon cancer [56]. In this study, all the five genes were shown to be dysregulated and have prognostic roles in HCC, indicating their close relations to HCC progression, consistent with previous studies [25, 57–59]. In addition, all of the five genes were also found to be significantly differentially expressed among HCC subtypes, presenting their heterogeneity in HCC samples, consistent with the differences among the HCC subtypes.

In the four HCC cell lines, the expressional differences of the key genes were also shown. At single-cell level, they all presented significant expressional variations among different hepatocyte subclusters, especially in primary HCC, which might account for superior malignant behaviors of a proportion of tumor cells in HCC. Besides, ADH4 and PON1 were also shown to be differentially expressed among the three hepatocyte subclusters in normal livers, which might be associated with HCC occurrence risk differences. Similarly, metastases in lymph nodes and portal vein tumor thrombus also presented their significant expressional variation of PON1 and NR0B1 in the hepatocyte subclusters, which might be associated with their different invasion capacity. At protein level, dysregulations of the genes were also confirmed and the significant negative correlation of ADH4 and PON1 with HCC proliferation and vascular invasion were shown, further indicating their close relation to HCC development and progression. In contrast, NR0B1 upregulation with positive correlation with microvascular invasion in HCC confirmed its positive expression in primary HCCs, metastatic lymph nodes, and portal vein tumor thrombus at single-cell level. In cervical squamous cell carcinoma and tongue squamous cell cancer, MMP10 was shown to be positively correlated with lymph node metastasis [60, 61]. Herein, we found the highest expression of MMP10 in the hepatocyte subclusters in metastatic lymph nodes and its close relation to lymph node metastasis was also indicated.

We also investigated the relationship of the five gene with the sensitivities of HCC cell lines to anticancer drugs. The negative correlations of ADH4, PON1, and PZP expression with the IC50s of the anti-cancer drugs indicated that the LSGs might affect the efficiency of these drugs in HCC. For MMP10 and NR0B1, their positive correlation or positive correlation trend with the anti-cancer drugs suggested HCC resistance to the drugs. These results uncovered the potential of these genes as indicators of HCC sensitivities to therapeutic drugs.

There were also limitations in our study. Firstly, the specific roles of the key genes during HCC occurrence and progression needed further study. Secondly, the clinical significance of the risk model needed to be validated in a broader population. Thirdly, single-cell RNA sequencing also had some technical limitations, such as dropout and bias in gene expression measurements, which may influence the accuracy of the conclusions. However, as multiple levels of analysis indicated the importance of the key genes for HCC, further study of them might provide valuable clues for HCC treatment.

## Conclusion

In summary, through systemic analyses of LSGs, we identified and validated three LSGs-based HCC subtypes with different prognosis, different stemness, different aging level, and different immune response. C1 subtype with low LSGs score and high immune score was shown to have a poor survival while C2 subtype with high LSGs score and immune score had a good survival. Although no significant survival difference between C2 HCC and C3 HCC was shown, C2 HCC presented higher immune score and stroma score than C3. These disparities between the HCC subtypes indicated the necessity of different treatment options for individual subtypes. We also constructed a five-gene prognostic signature for HCC survival and the effective performance was shown. The five genes were heterogeneously expressed in HCC cell lines and hepatocyte subclusters in primary HCC. There dysregulations were confirmed at protein level. Furthermore, their associations with HCC response to anti-cancer drugs were uncovered. The results indicated that the LSGs-based HCC classification and the five-gene risk model were conductive to HCC stratification and risk prediction. The study might provide novel clues for more effective treatment options and promote HCC precision treatment. Further clinical studies and experiments are necessary to help the clinical translation and application of the key genes.

### Supplementary Information

**Additional file 1: Table S1. **Clinical characters of the patients in TCGA-HCC dataset and ICGC-HCC dataset. **Table S2. **Expressional differences between HCC and normal liver tissues. **Table S3. **The LSGs differences among different HCC subtypes. **Table S4. **LSGs methylation comparisons among HCC subtypes. **Table S5. **LSGs expression comparisons among HCCs with different risk factors. **Table S6. **HCC subtype-specific genes. **Table S7. **Predication of HCC subtype with SVM method in TCGA-HCC dataset. **Table S8. **HCC subtype predication with SVM model in ICGC-HCC samples. **Table S9. **HCC subtype predication of TCGA-HCC samples with NTP method. **Table S10. **HCC subtype predication of ICGC-HCC samples with NTP method. **Table S11. **Prognostic effects of LSGs and HCC subtype-specific genes. **Table S12. **Hepatocyte subcluster proportions in different tissues. **Table S13. **The correlations of the key genes with the IC50s of anti-cancer drugs in HCC cell lines.

** Additional file 2:**  **Figure S1. **The differences of immune cell infiltrations among different HCC subtypes. **Figure S2. **Gene expression comparisons between different HCC subtypes in TCGA-HCC dataset. **Figure S3. **The significant terms of HCC subtype-specific DEGs in GO enrichment. **Figure S4. **The top-50 CpG site of the LSGs with significant differences among the HCC subtypes. **Figure S5. **The representative CpG sites of the LSGs with significant difference among the HCC subtypes. **Figure S6. **The risk factor composition comparisons among different HCC subtypes. **Figure S7. **The survival differences between low- and high-risk patients in TCGA-HCC (A) and ICGC-HCC (B) datasets. **Figure S8. **The prognostic effects of risk score, liver fibrosis, and serum AFP on HCC OS. **Figure S9. **Expressional differences of the key genes between different HCC subtypes. **Figure S10. **The cell clusters in tumor and normal tissues (A) and their marker gene expressions (B). **Figure S11. **Positive rate comparisons of the key genes among different cell types. **Figure S12. **Expressional heterogeneity of the key genes among hepatocyte subclusters in different tissues.

## Data Availability

All data and materials are provided in this article and the additional files, which can be available publicly.

## Abbreviations

LSGs: Liver specific genes
HCC: Hepatocellular carcinoma
TCGA: The Cancer Genome Atlas
ICGC: International Cancer Genome Consortium
TSGs: Tissue-specific genes
EGFR: Epidermal growth factor receptor
CEA: Carcinoembryonic antigen
PSM: Prostate-specific membrane antigen
scRNA: Single cell RNA
TiGER: Tissue-specific gene expression and regulation
HPA: Human protein atlas
OS: Overall survival
DFS: Disease-free survival
MSigDB: Molecular Signatures Database
GSVA: Gene set variation analysis
TCIA: The cancer immunome atlas
DEGs: Differentially expressed genes
logFC: log_2_(fold change)
GO: Gene ontology
GSEA: Gene set enrichment analysis
SVM: Support vector machine
NTP: Nearest template prediction
LASSO: Least absolute shrinkage and selection operator
ROC: Receiver operating characteristic
CCLE: Cancer Cell Line Encyclopedia
IC50s: Half maximal inhibitory concentrations
FDR: False discovery rate
