# Supplementary material for: Signature construction and molecular subtype identification based on liver-specific genes for prediction of prognosis, immune activity, and anti-cancer drug sensitivity in hepatocellular carcinoma
Source: Cancer Cell Int. 2024 Feb 19;24:78. doi: 10.1186/s12935-024-03242-3 (PMC10875877; doi:10.1186/s12935-024-03242-3)
Supplement: Supplementary file 2 — Additional file 2: Figure S1. The differences of immune cell infiltrations among different HCC subtypes. Figure S2. Gene expression comparisons between different HCC subtypes in TCGA-HCC dataset. Figure S3. The significant terms of HCC subtype-specific DEGs in GO enrichment. Figure S4. The top-50 CpG site of the LSGs with significant differences among the HCC subtypes. Figure S5. The representative CpG sites of the LSGs with significant difference among the HCC subtypes. Figure S6. The risk factor composition comparisons among different HCC subtypes. Figure S7. The survival differences between low- and high-risk patients in TCGA-HCC (A) and ICGC-HCC (B) datasets. Figure S8. The prognostic effects of risk score, liver fibrosis, and serum AFP on HCC OS. Figure S9. Expressional differences of the key genes between different HCC subtypes. Figure S10. The cell clusters in tumor and normal tissues (A) and their marker gene expressions (B). Figure S11. Positive rate comparisons of the key genes among different cell types. Figure S12. Expressional heterogeneity of the key genes among hepatocyte subclusters in different tissues. [file 12935_2024_3242_MOESM2_ESM.docx]

**Supplementary figures**


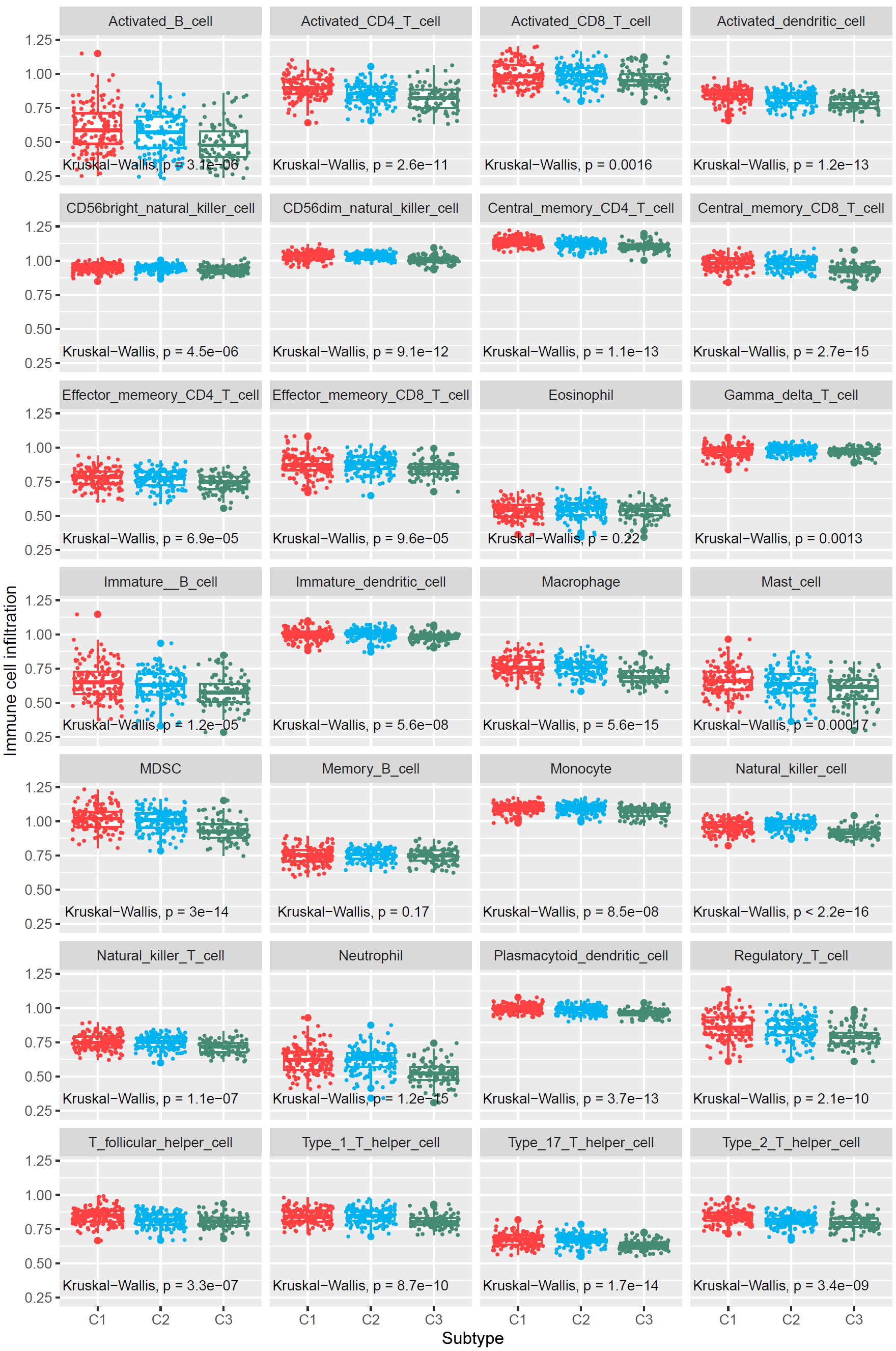


**Figure S1.** The differences of immune cell infiltrations among different HCC subtypes.

Figure S2. Gene expression comparisons between different HCC subtypes in TCGA-HCC dataset.


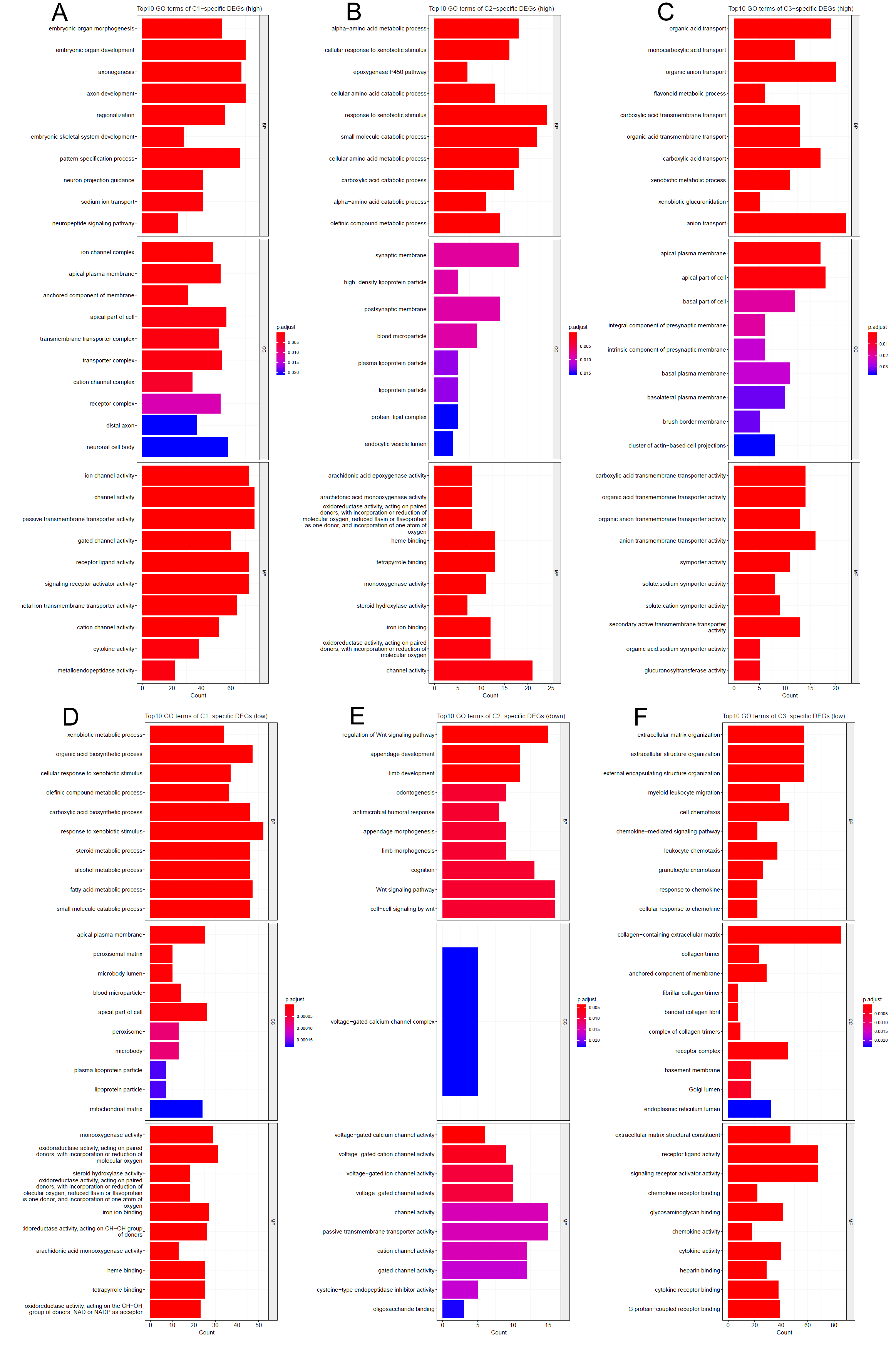


Figure S3. The significant terms of HCC subtype-specific DEGs in GO enrichment. (A-C) The GO enrichments of the higher expressed C1-, C2, and C3-specific DEGs, respectively. (D-F) The GO enrichments of the lower expressed C1-, C2, and C3-specific DEGs, respectively. BP, biological process; CC, cell component; MF, molecular function.


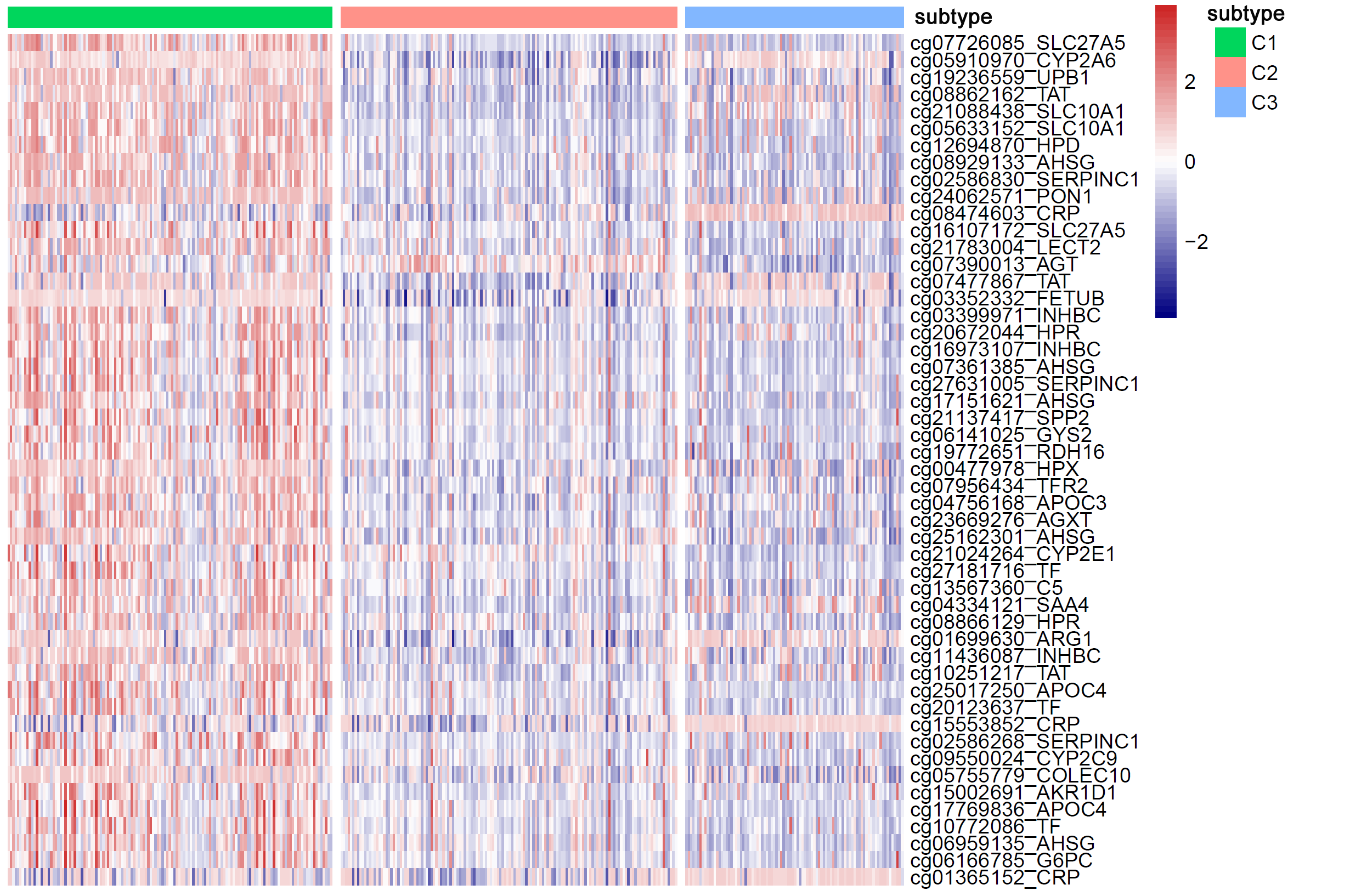


Figure S4. The top-50 CpG site of the LSGs with significant differences among the HCC subtypes. LSGs, liver-specific genes.


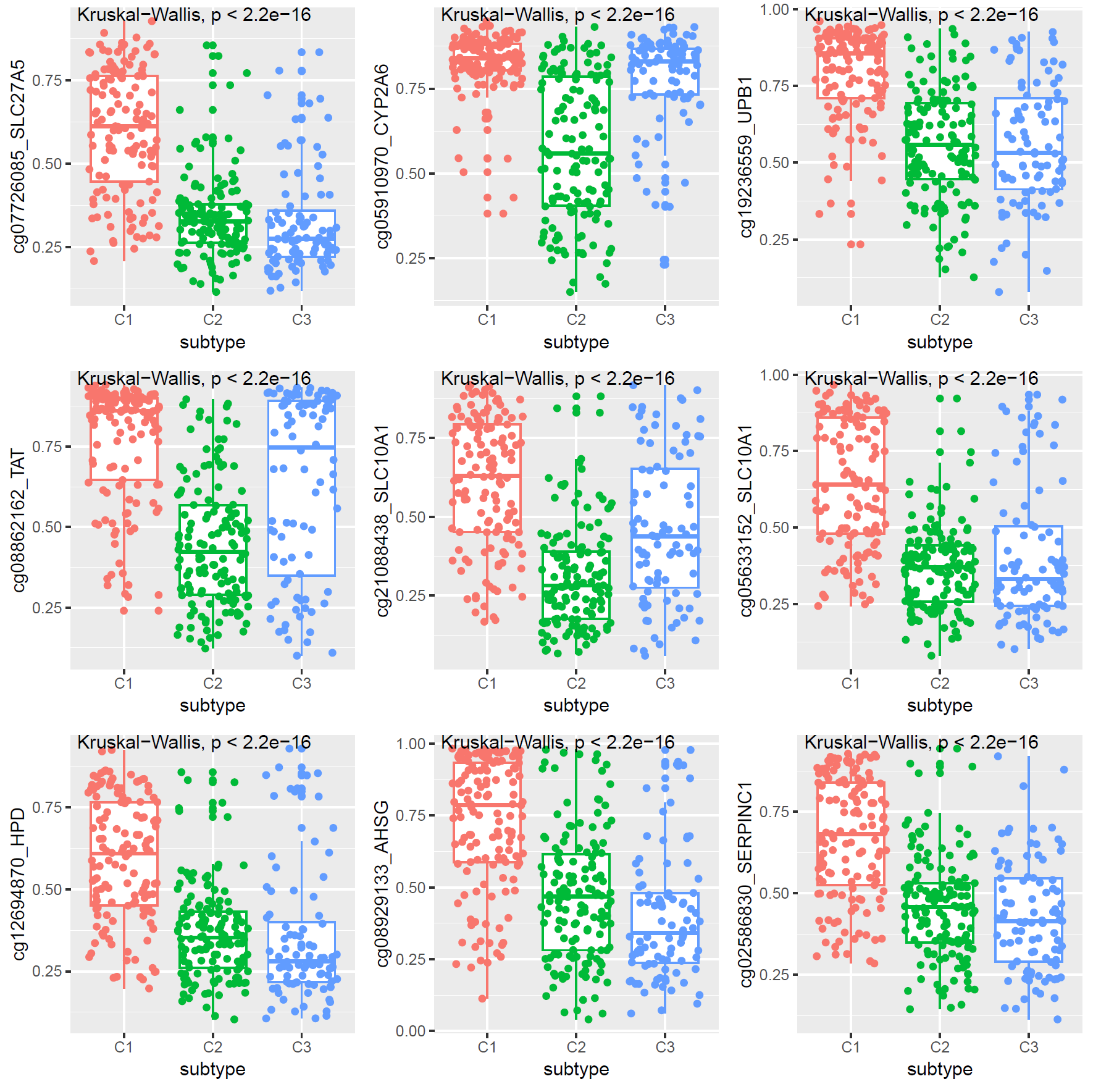


Figure S5. The representative CpG sites of the LSGs with significant difference among the HCC subtypes. Kruskal-Wallis test was used for comparisons and *p* < 0.05 was considered significant.


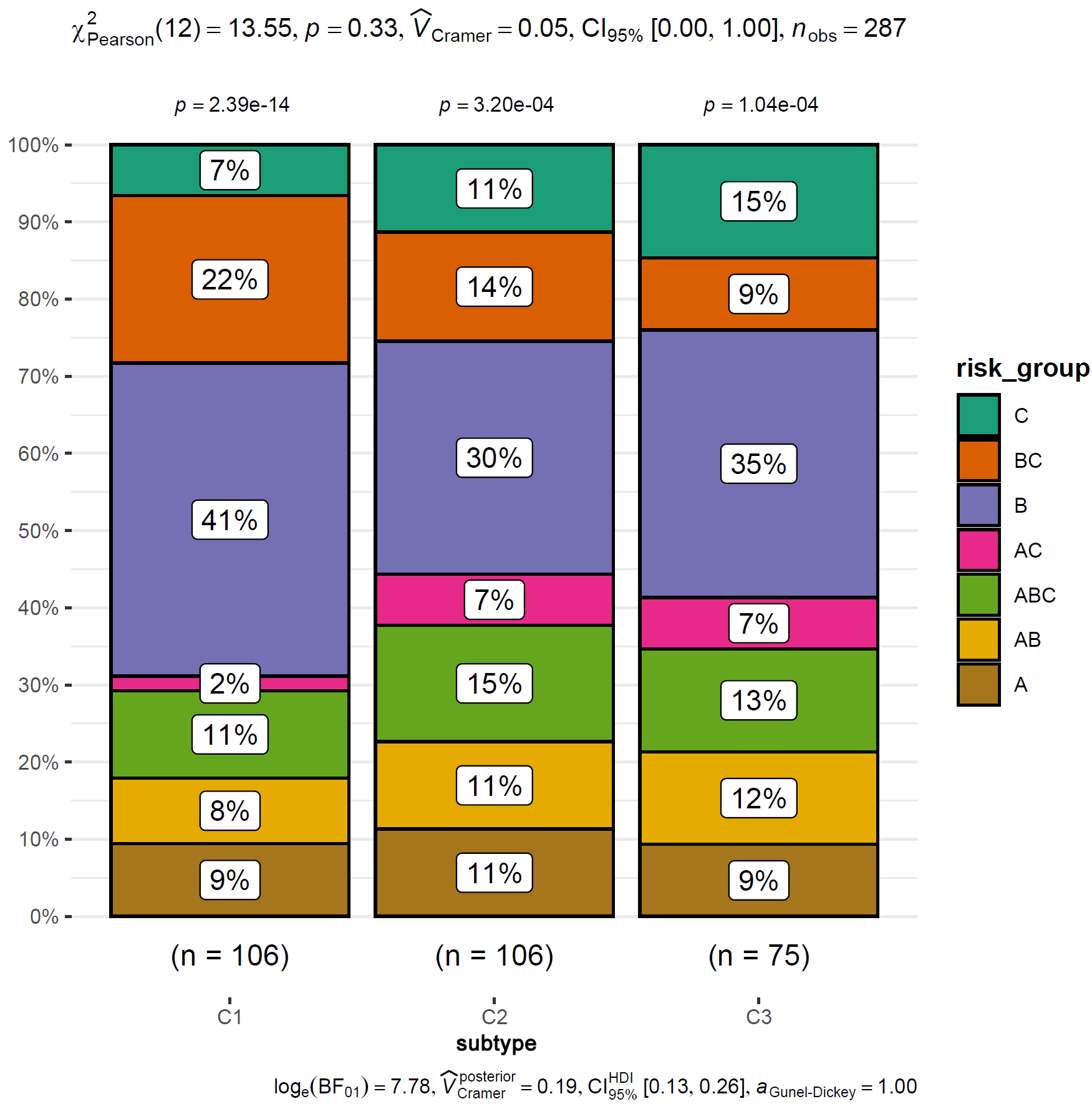


Figure S6. The risk factor composition comparisons among different HCC subtypes. For the risk-groups, A, B, and C represented alcohol assumption, HBV hepatitis, and HCV hepatitis, respectively. AB: alcohol assumption and HBV hepatitis; AC: alcohol assumption and HCV hepatitis; BC: HBV hepatitis and HCV hepatitis; ABC: alcohol assumption, HBV hepatitis, and HBV hepatitis. Only the risk groups with sample size larger than ten were included for analysis. Char-square test was used for comparisons and *p* < 0.05 was considered significant.


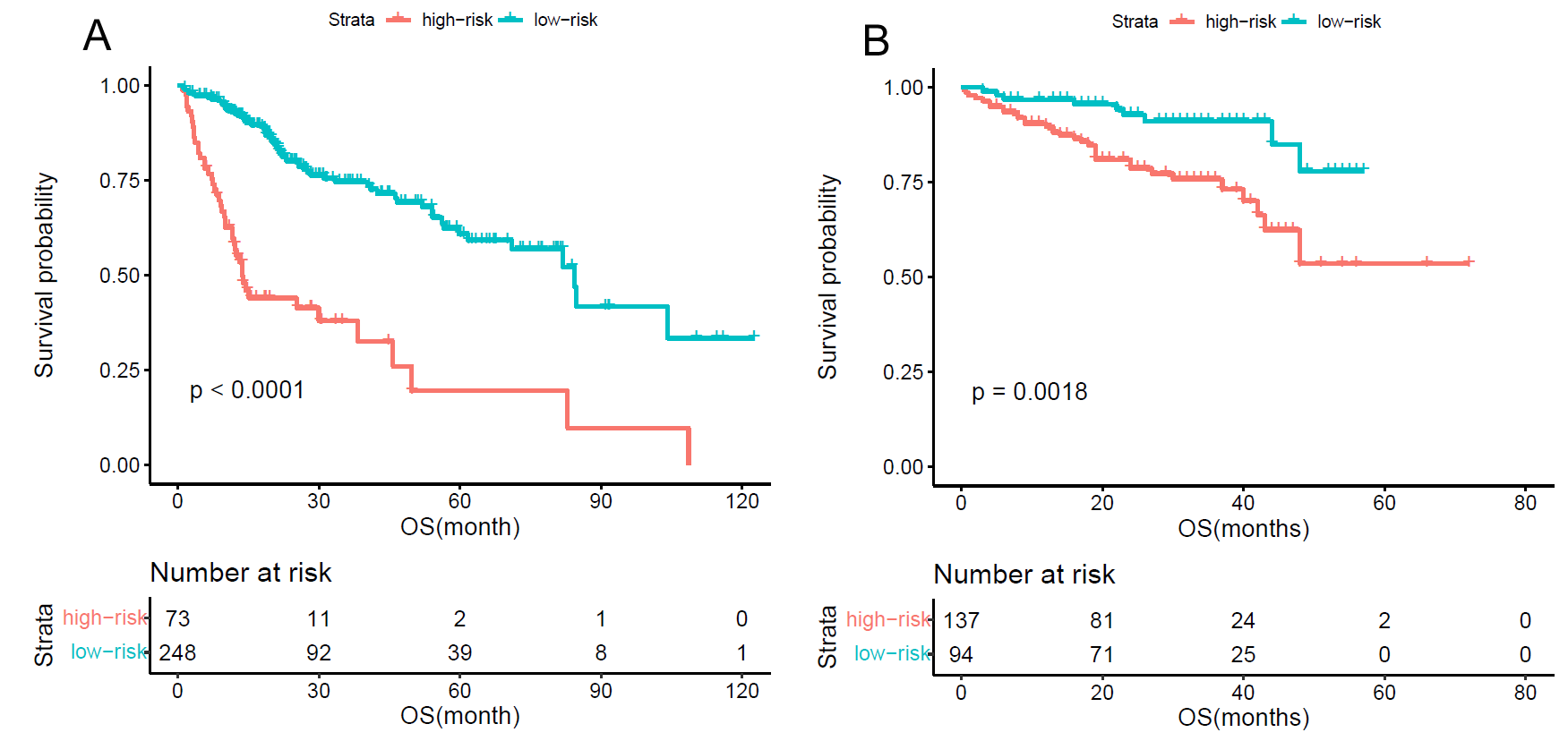


Figure S7. The survival differences between low- and high-risk patients in TCGA-HCC (A) and ICGC-HCC (B) datasets.

Figure S8. The prognostic effects of risk score, liver fibrosis, and serum AFP on HCC OS. OS, overall survival. Multi-variable Cox regression analysis was used and *p* < 0.05 was considered significant. For the analysis, serum AFP level was log_2_(x) transformed.


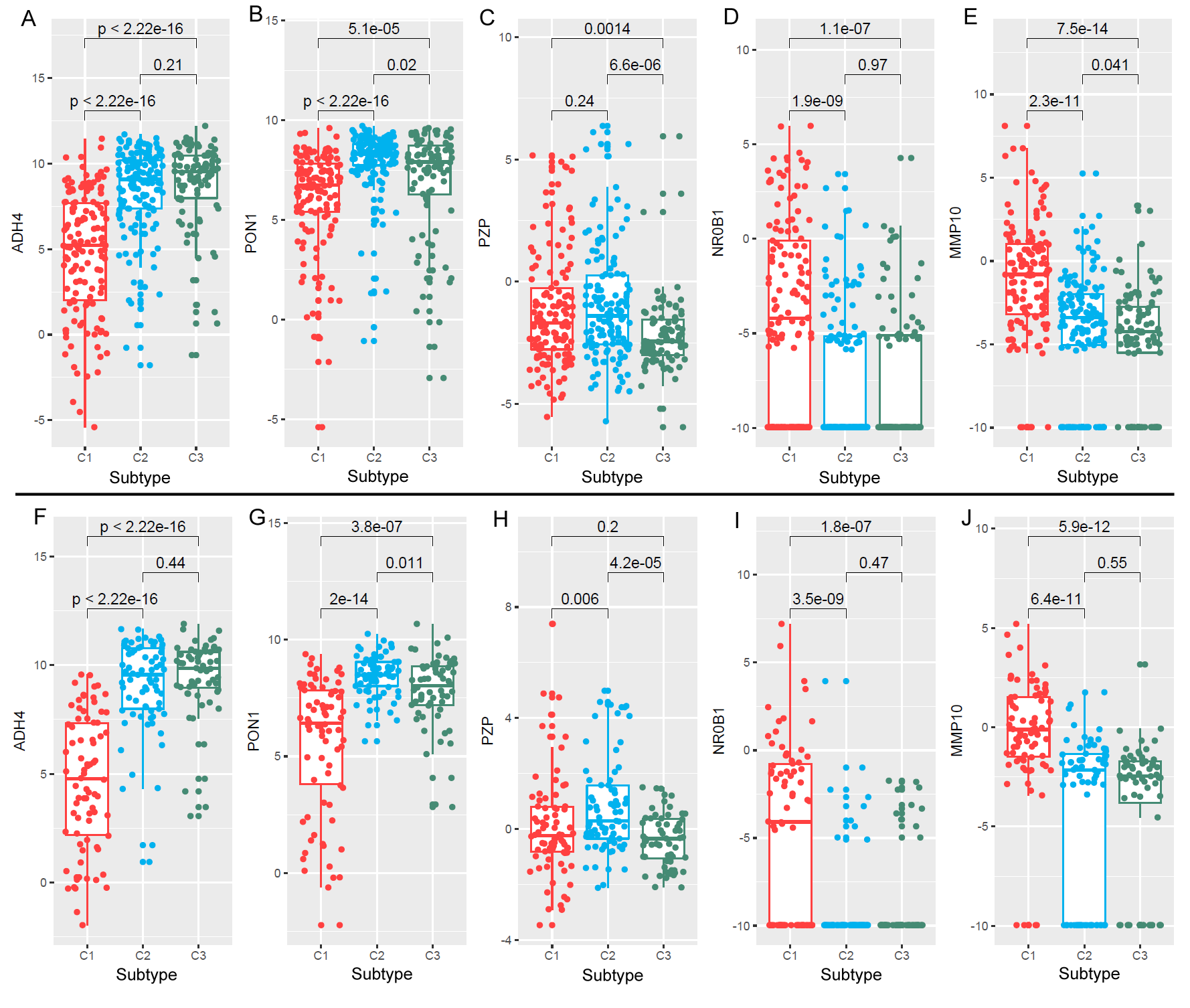


**Figure S9.** Expressional differences of the key genes between different HCC subtypes. (A-E) Expression comparisons of ADH4, PON1, PZP, NR0B1, and MMP10 between different HCC subtypes (clusters) in TCGA-HCC dataset. (F-J) Expression comparisons of ADH4, PON1, PZP, NR0B1, and MMP10 between different HCC subtypes (clusters) in ICGC-HCC dataset.


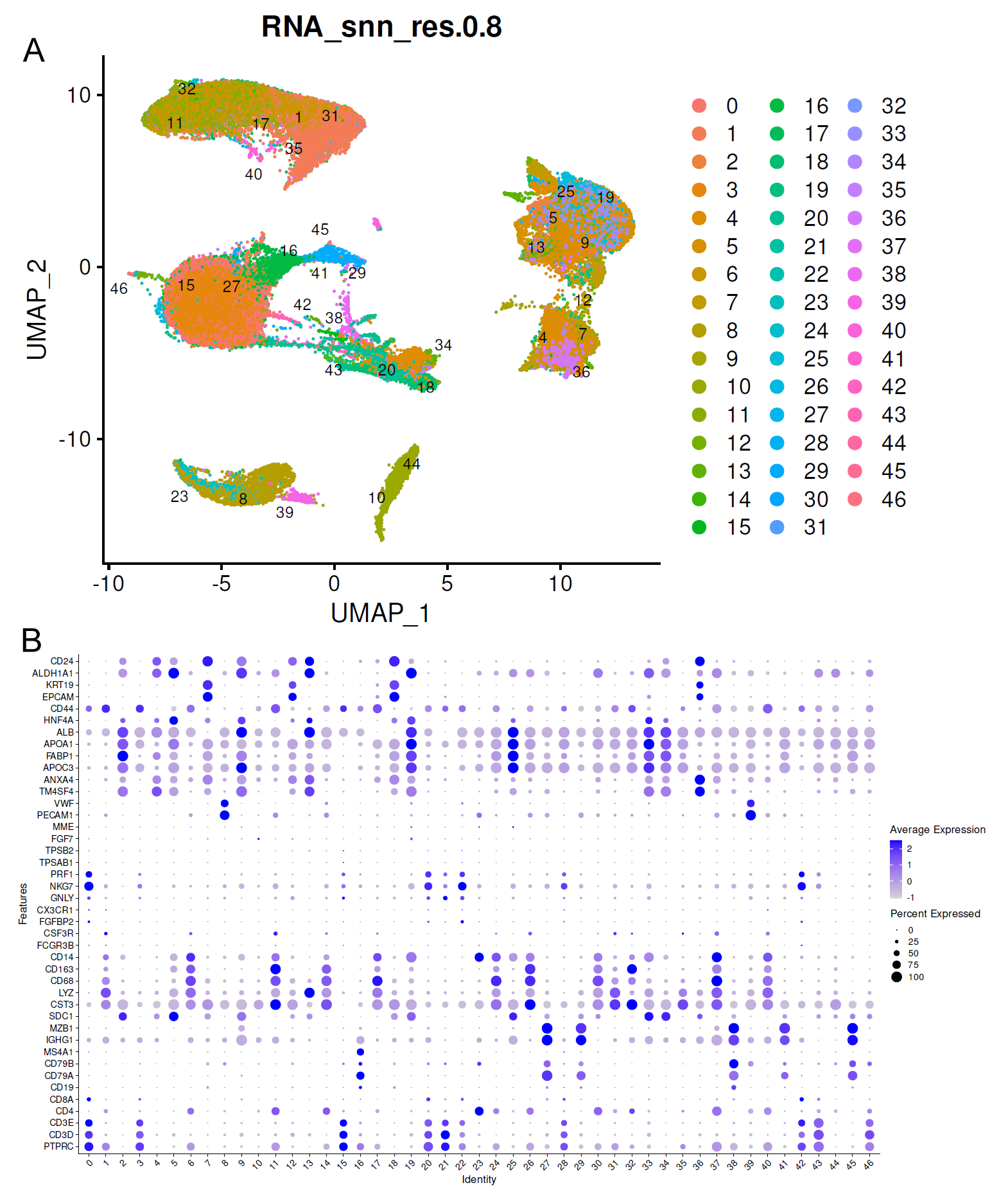


**Figure S10.** The cell clusters in tumor and normal tissues (A) and their marker gene expressions (B).


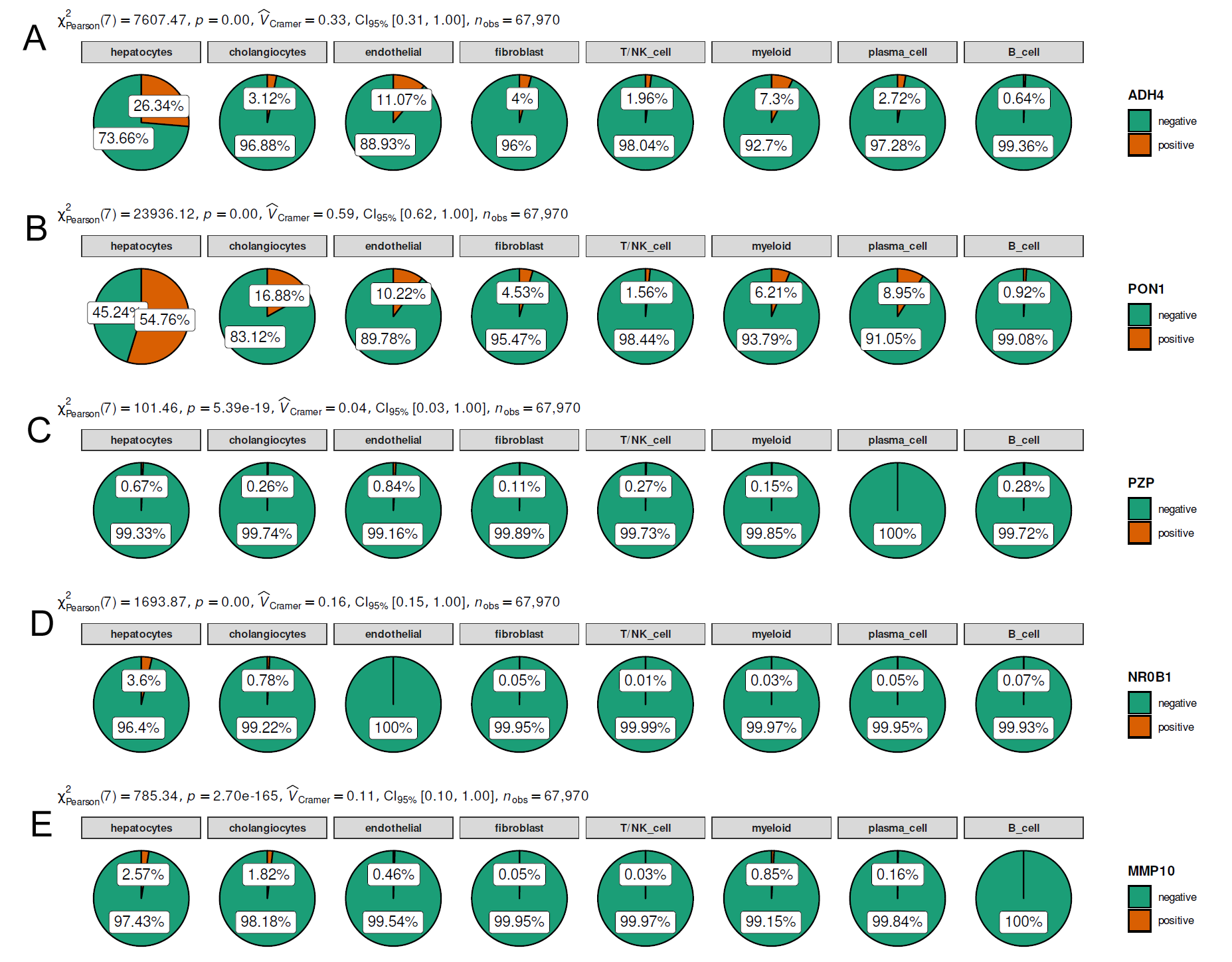


**Figure S11**. Positive rate comparisons of the key genes among different cell types. (A-E) There were significant differences of the positive expression rate of ADH4, PON1, PZP, NR0B1, and MMP10 in different cell types. Chi-square test was used and *p* < 0.05 was considered significant.


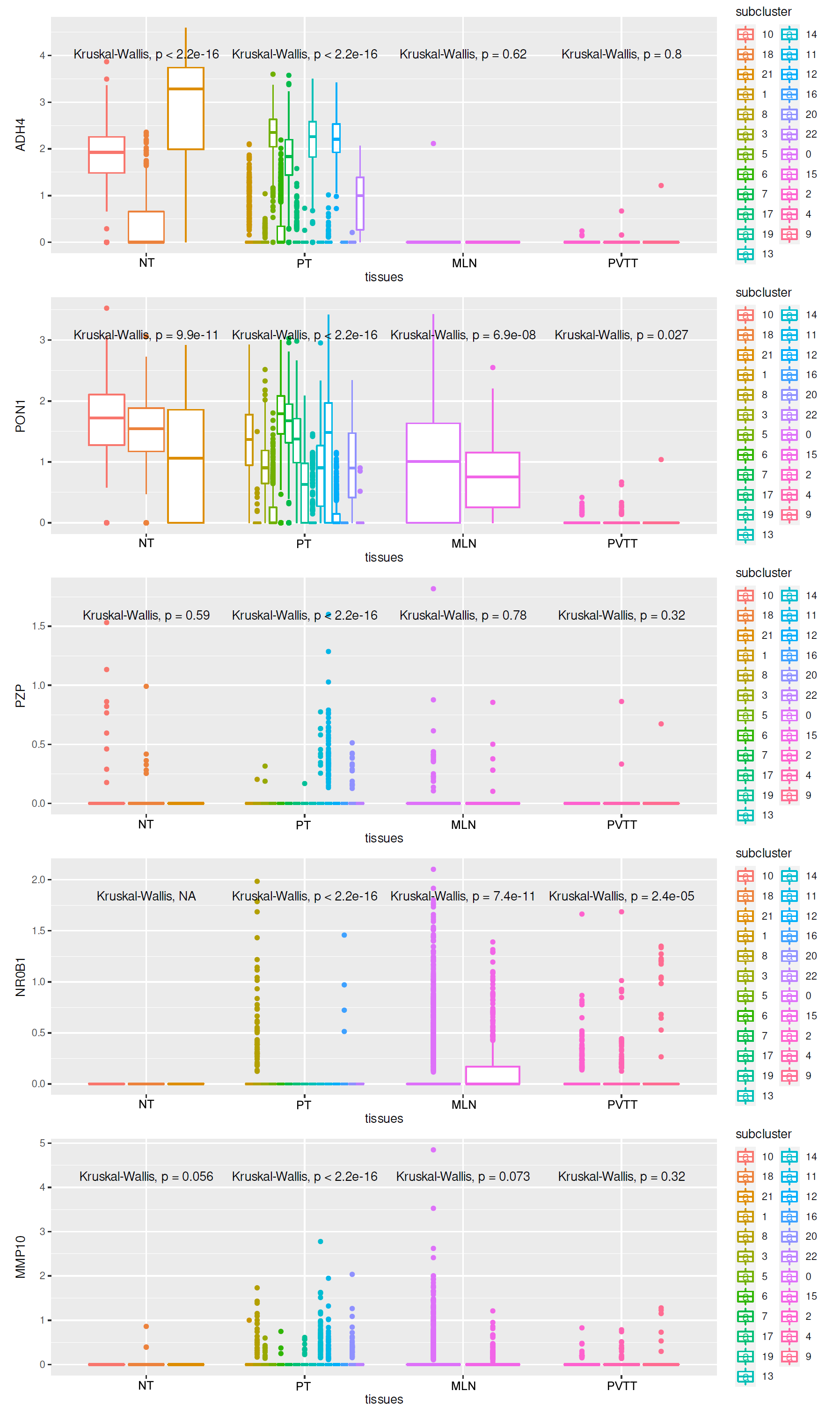


**Figure S12**. Expressional heterogeneity of the key genes among hepatocyte subclusters in different tissues. NT, non-tumor liver; PT, primary tumor; MLN, metastatic lymph nodes; PVTT, portal vein tumor thrombus. Kruskal-Wallis test was used for comparisons and *p* < 0.05 was considered significant.
